# Rh–Sb Nanoclusters: Synthesis, Structure, and Electrochemical Studies of the Atomically Precise [Rh_20_Sb_3_(CO)_36_]^3–^ and [Rh_21_Sb_2_(CO)_38_]^5–^ Carbonyl Compounds

**DOI:** 10.1021/acs.inorgchem.9b03135

**Published:** 2020-03-24

**Authors:** Cristina Femoni, Tiziana Funaioli, Maria Carmela Iapalucci, Silvia Ruggieri, Stefano Zacchini

**Affiliations:** † Dipartimento di Chimica Industriale “Toso Montanari”, Università di Bologna, Viale del Risorgimento 4, 40136 Bologna, Italy; ‡ Dipartimento di Chimica e Chimica Industriale, Università di Pisa, Via Moruzzi 13, 56124 Pisa, Italy

## Abstract

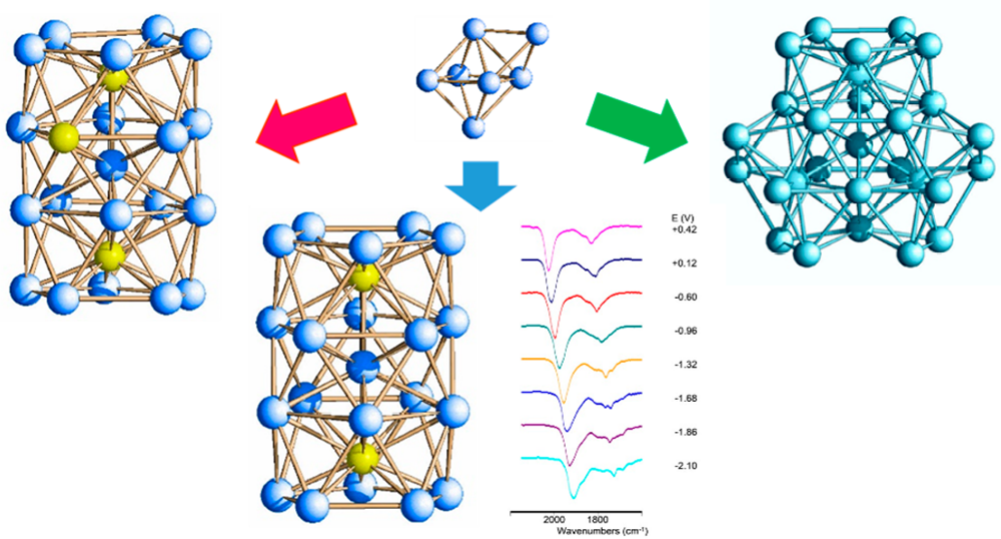

The reactivity of [Rh_7_(CO)_16_]^3–^ with SbCl_3_ has been deeply investigated with the aim of finding a new approach to prepare atomically precise metalloid clusters. In particular, by varying the stoichiometric ratios, the reaction atmosphere (carbon monoxide or nitrogen), the solvent, and by working at room temperature and low pressure, we were able to prepare two large carbonyl clusters of nanometer size, namely, [Rh_20_Sb_3_(CO)_36_]^3–^ and [Rh_21_Sb_2_(CO)_38_]^5–^. A third large species composed of 28 metal atoms was isolated, but its exact formulation in terms of metal stoichiometry could not be incontrovertibly confirmed. We also adopted an alternative approach to synthesize nanoclusters, by decomposing the already known [Rh_12_Sb(CO)_27_]^3–^ species with PPh_3_, willing to generate unsaturated fragments that could condense to larger species. This strategy resulted in the formation of the lower-nuclearity [Rh_10_Sb(CO)_21_PPh_3_]^3–^ heteroleptic cluster instead. All three new compounds were characterized by IR spectroscopy, and their molecular structures were fully established by single-crystal X-ray diffraction studies. These showed a distinct propensity for such clusters to adopt an icosahedral-based geometry. Their characterization was completed by ESI-MS and NMR studies. The electronic properties of the high-yield [Rh_21_Sb_2_(CO)_38_]^5–^ cluster were studied through cyclic voltammetry and *in situ* infrared spectroelectrochemistry, and the obtained results indicate a multivalent nature.

## Abstract 2

The reactivity of [Rh_7_(CO)_16_]^3−^ with SbCl_3_ has been deeply investigated as a new approach to prepare atomically precise metal nanoparticles. By varying the reaction conditions, we obtained three large carbonyl nanoclusters, [Rh_20_Sb_3_(CO)_36_]^3−^, [Rh_21_Sb_2_(CO)_38_]^5−^, and [Rh_28−*x*_Sb_*x*_(CO)_44_]^6−^, and the lower-nuclearity [Rh_10_Sb(CO)_21_PPh_3_]^3−^ species. They have all been characterized through X-ray diffraction, IR spectroscopy, and other techniques based on their specific nature. Spectroelectrochemical studies on [Rh_21_Sb_2_(CO)_38_]^5−^ unravelled its multivalent nature.

## Introduction

Transition-metal carbonyl clusters have been deeply studied over the last decades, and lately, the literature has been enriched with growing numbers of new high-nuclearity species in the nanometer regime,^1^ to the point that it is now possible to insert carbonyl clusters in the field of molecular nanoparticles. Moreover, one of the most captivating aspects of those compounds is their atomic precision.^2^ In fact, even though they can reach a nanometer size, they still possess a molecular nature, so their structure and composition can be unambiguously unravelled.

Nowadays, the tuning of their size and composition has also become a feasible reality, and it is possible to prepare the desired nanoclusters, both homo- and heterometallic, in order to exploit them, for instance, as catalyst precursors for application in both homogeneous and heterogeneous reactions.^3^

In the specific field of Rh carbonyl clusters, where we have been active for a few years, several homometallic species of high nuclearity are reported in the literature,^4,5^ partly thanks to the high energies of the Rh–Rh and Rh-CO bonds^6,7^ that favor the cluster growth. In addition, Rh can be combined with other elements to obtain heterometallic compounds, and the heteroatom(s) can be found in either peripheral^8^ or interstitial positions,^9^ or in both.^10^ It has been experimentally demonstrated that when the heteroatom is interstitially lodged it imparts more stability to the metal skeleton.^11^ As a matter of fact, there are several stable species containing light p elements, such as C^12^ or N,^13^ as well as heavier ones such as P,^14^ S,^15^ or even Ge,^16,2^ Sn,^17,18^ Sb,^19,20^ and Bi.^10^ In the case of the latter heavier metals, all those Rh–E systems share the icosahedral [Rh_12_E(CO)_27_]^*n*−^ species (*n* = 4 when E = Ge, Sn; *n* = 3 when E = Sb, Bi). Beyond that, they do take different paths and give rise to different heterometallic nanometer compounds.

With the purpose of further deepening the chemistry of heterometallic carbonyl clusters and testing the possibility of synthesizing new nanoparticles with less conventional methods, we extended the investigation of the Rh–Sb system. Beside its academic relevance, it could be interesting to study the combination of those two elements for applications in other fields. For instance, within studies of catalytic degradation of pollutants, the co-doping effect of rhodium and antimony on TiO_2_ reduces the band-gap energy and lead to a better photocatalytic activity if compared with a non-doped TiO_2_ system.^21^ Moreover, it could be interesting to explore the electronic properties of large clusters in terms, for instance, of their possibility to act as nanocapacitors and be able to reversibly accept and release electrons. Those multivalent features have been previously observed in similar species possessing specific ad hoc conditions.^22^

Currently, the unique Rh–Sb homoleptic carbonyl clusters reported in the literature are the icosahedral [Rh_12_Sb(CO)_27_]^3–^ species, obtained for the first time by Vidal’s group^19^ by exploiting high temperatures and elevated CO pressures, and its coordinatively and electronically unsaturated [Rh_12_Sb(CO)_24_]^4–^ derivative.^20^ However, if we take into consideration the combination of Sb with other transition metals, then we can find many examples of large Ni carbonyl species where Sb differently coordinates to the metal framework. For instance, in [Ni_15_Sb(CO)_24_]^2–^,^23^ the heteroatom is inside the metal cavity, while in [Ni_11_Sb_2_(CO)_18_]^3–^^23^ and [Ni_10_(SbR)_2_(CO)_18_]^2–^ (R = Me, Et, *i*Pr, *t*-Bu, and *p*-FC_6_H_4_),^24^ the two Sb atoms cap the external pentagonal faces. Finally, in [Ni_31_Sb_4_(CO)_40_]^6–^,^25^ the four antimony atoms are semi-interstitially lodged. Other compounds are reported within the Os–Sb and Ru–Sb systems, but with lower nuclearity. In the neutral Os_3_(SbPh_2_)_2_(CO)_10_^26^ and Ru_6_(SbPh_2_)_2_(CO)_20_,^27^ the SbPh_2_ groups act as bridging ligands on the metal surface, while in both Os_6_(μ_5_-Sb)(μ-H)_2_(μ-SbPh_2_)(μ_3_,η^2^-C_6_H_4_)(CO)_17_ and Ru_6_(μ_5_-Sb)(μ-H)_3_(SbPh_3_)(CO)_18_,^28^ the naked Sb atom connects two cluster fragments.

In order to synthesize new Rh–Sb nanoclusters we mainly exploited the redox-condensation method, which proved to be very effective in the past for similar systems, by reacting the preformed [Rh_7_(CO)_16_]^3–^ cluster with halides of Sb^3+^ under different operative conditions (stoichiometric ratio, solvent, atmosphere). This led us to isolate and fully characterize two new different cluster compounds, namely [Rh_20_Sb_3_(CO)_36_]^3–^ and [Rh_21_Sb_2_(CO)_38_]^5–^, all of nanometer size. We also isolated a third large species, tentatively formulated as [Rh_25_Sb_3_(CO)_44_]^6–^ on the basis of the analyses performed via electrospray ionization mass spectrometry (ESI-MS) and energy dispersive X-ray spectrometry (EDS) through Scanning Electron Microscopy (SEM), coupled with the X-ray diffraction data. However, the latter were not of sufficient quality to confirm the stoichiometric metal ratio, albeit good enough to determine its metal structure, so we reformulated it as [Rh_28–*x*_Sb_*x*_(CO)_44_]^6–^. Furthermore, we tried to obtain new compounds through disaggregation of a cluster precursor, namely [Rh_12_Sb(CO)_27_]^3–^, so to form unstable unsaturated fragments that could, in turn, condense giving larger species. Instead, we obtained the lower-nuclearity [Rh_10_Sb(CO)_21_PPh_3_]^3–^ cluster stabilized by the phosphine ligand. All clusters were characterized by infrared (IR) spectroscopy, and their molecular structures were determined by single-crystal X-ray diffraction analysis. The [Rh_10_Sb(CO)_21_PPh_3_]^3–^ heteroleptic cluster was also characterized through ^31^P NMR, while the three larger compounds were analyzed by ESI-MS. Finally, the [Rh_21_Sb_2_(CO)_38_]^5–^ cluster was investigated through electrochemical and *in situ* Fourier transform infrared (FT-IR) spectroelectrochemical studies, and the obtained data point to the existence of a rich redox chemistry and multivalent nature, as inferred by the comparison with analogous results obtained for similar clusters. However, the low-intensity current showed during the cyclic voltammetry (CV) study, and the absence of some isosbestic points in the spectroelectrochemistry, prevented us from directly assigning the number of the electrons exchanged in each redox step and, consequently, the charge and number of the oxidation states in which cluster **3** can stably exist.

## Results and Discussion

### Synthesis and Spectroscopic Characterization of the New Heterometallic [Rh_20_Sb_3_(CO)_36_]^3–^, [Rh_28–*x*_Sb_*x*_(CO)_44_]^6–^, [Rh_21_Sb_2_(CO)_38_]^5–^, and [Rh_10_Sb(CO)_21_PPh_3_]^3–^ Carbonyl Nanoclusters

In order to synthesize new Rh–Sb carbonyl nanoclusters we first employed the so-called redox condensation method, which was initially described by Hieber and Schubert,^29^ and later on exploited by Chini,^30^ by reacting the [Rh_7_(CO)_16_]^3–^^31^ cluster precursor with a salt of Sb^3+^ in different reaction conditions (stoichiometric ratio, atmosphere, and solvent).

In particular, we reacted [Rh_7_(CO)_16_]^3–^ and SbCl_3_ in acetonitrile under CO atmosphere with a final molar ratio of 1:1.15. After a few hours, the final mixture showed a different IR spectrum (2068 (s), 2024 (vs), 1991 (s), and 1824 (ms) cm^–1^) from that of the known icosahedral compound. We dried the solution under vacuum and washed the residue with water to remove the inorganic salts and with ethanol to subtract the [Rh(CO)_2_Cl_2_]^−^ complex (responsible for the ν_CO_ absorptions at 2068 (vs) and 1991 (vs) cm^–1^). After a further washing with THF, we extracted in acetone a species showing an unknown IR spectrum (2030 (vs) and 1830 (ms) cm^–1^; these signals were detected in the reaction mixture but with a slight downshift owing to the solvent effect). We layered *n*-hexane onto the solution in order to obtain suitable crystals for a structural analysis, and the X-ray diffraction experiment allowed us to characterize the new [Rh_20_Sb_3_(CO)_36_]^3–^ nanocluster (**1**) in its [NEt_4_]^+^ salt (yield 60% based on Rh). Its molecular structure is discussed in the next section. The same synthesis, but in acetone rather than acetonitrile, led to a product with an IR spectrum similar to that of cluster **1**; however, it was not possible to confirm it owing to lack of crystalline samples. We evaluated the possibility of synthesizing other new Rh–Sb clusters under CO atmosphere by using the same strategy, but further additions of Sb^3+^ to the homometallic cluster precursor beyond 1.5 equiv only lowered the yield of **1**, owing to its partial degradation in favor of the [Rh(CO)_2_Cl_2_]^−^ complex. Cluster **1** was also characterized by ESI-MS spectrometry (see the Experimental Section and the Supporting Information).

Thanks to its fairly high yield, we could perform some reactivity studies on this new cluster. An acetone solution of **1**[NEt_4_]_3_ was refluxed under N_2_ atmosphere to test its stability at high temperature. After 2 h, the solution showed a different IR spectrum, consistent with that of the known saturated icosahedral species. The degradation of [Rh_20_Sb_3_(CO)_36_]^3–^ in favor of the [Rh_12_Sb(CO)_27_]^3–^ cluster was confirmed through the ESI-MS analysis; in fact, the spectrum exhibited three groups of peaks starting at 1121, 1028, and 704 *m*/*z*, assigned to the {[Rh_12_Sb(CO)_27–26–25_][NEt_4_]}^2–^, [Rh_12_Sb(CO)_25–24–23–22–21_]^2–^, and [Rh_12_Sb(CO)_27–26–25–24_]^3–^ ions, respectively.

In previous studies, it was experimentally demonstrated that under N_2_ atmosphere the [Rh_12_Sb(CO)_27_]^3–^ cluster gave rise to the coordinatively and electronically unsaturated [Rh_12_Sb(CO)_24_]^4–^ species. This result encouraged us to investigate the synthesis of new Rh–Sb carbonyl clusters by working under inert nitrogen atmosphere. More specifically, we carried out the reaction between [Rh_7_(CO)_16_]^3–^ and SbCl_3_ in acetonitrile under N_2_. However, we stopped the addition of the Sb^3+^ salt after 0.7 equiv, as opposed to 1.15, because of the total disappearance of the ν_CO_ absorptions of [Rh_7_(CO)_16_]^3–^ in favor of new signals. At the end of the reaction, the extraction in acetone solubilized a new cluster (**2**), together with traces of a sparingly soluble new species. The latter unknown carbonyl compound (an assignment based on the sole IR analysis) was isolated in the subsequent extraction in acetonitrile, which showed a clean spectrum with the same signals; unfortunately, because of its very low yield, it was not possible to identify it. Conversely, cluster **2** was crystallized as salt of [NEt_4_]^+^ by layering *n*-hexane onto the acetone solution, and its metal structure was determined by single-crystal X-ray diffraction (see the next section). However, the quality of the obtained crystals was rather poor, so the data output was problematic. Any attempt to prepare better crystals by changing the counterion or the crystallization solvent did not succeed. Therefore, the formulation that could be derived from the crystallographic data was [Rh_28–*x*_Sb_*x*_(CO)_44_]^6–^, with an uncertainty on the metal ratio. In order to establish the Rh/Sb stoichiometry, we performed the EDS analysis on one crystal of **2**. The sample was mapped in different areas and the atomic Rh/Sb ratio derived from the analysis pointed toward a 25:3 value, respectively, being the mean atomic percentages of Rh and Sb in the crystal equal to 91.8 and 8.2%, respectively (see Table S2). To further substantiate the cluster characterization, we carried out an ESI-MS analysis on a sample on which we had performed a cation metathesis, in the attempt to obtain better quality crystals. In spite of the residual presence of another species (see the following section), the spectrum (see the Supporting Information) shows peaks that could be assigned to the following ions: {[Rh_25_Sb_3_(CO)_44_][NMe_4_]_2_}^3–^, {[Rh_25_Sb_3_(CO)_44_][NMe_4_]}^3–^, and {[Rh_25_Sb_3_(CO)_42_]}^3–^, alongside with other signals due to their CO loss. Even though all experimental results indicate that cluster **2** could be formulated as [Rh_25_Sb_3_(CO)_44_]^6–^, the crystallographic data are still not of sufficient quality to undoubtedly elaborate on its metal composition. Nonetheless, we can confidently affirm that cluster **2** consists of 28 metal atoms and possesses an icosahedral-based metal geometry (see the next section), therefore it may be indeed indicated as [Rh_28–*x*_Sb_*x*_(CO)_44_]^6–^

At this point, we changed another parameter in the operative conditions and conducted the reaction between [Rh_7_(CO)_16_][NEt_4_]_3_ and SbCl_3_ in acetone instead of acetonitrile to facilitate the separation of possible insoluble products in such solvent. We maintained the nitrogen atmosphere and reached a final stoichiometric Rh_7_/Sb^3+^ ratio of 1:0.8. Again, the end of the reaction was dictated by the disappearance of the IR signals of the cluster precursor. As expected, some insoluble residue was found in the mother solution, and once dissolved in acetonitrile, it presented a similar IR spectrum to the unknown cluster isolated in low yields during the synthesis of **2**. The layering of di-isopropyl ether onto the acetonitrile solution allowed us to obtain crystals suitable for X-ray analysis, and the cluster was identified as [Rh_21_Sb_2_(CO)_38_]^5–^ (**3**) in its [NEt_4_]^+^ salt. Its molecular structure is illustrated in the next section. As for the initial solution in acetone, its workup allowed us to isolate the usual [Rh(CO)_2_Cl_2_]^−^ complex in ethanol and THF, and the already known unsaturated compound [Rh_12_Sb(CO)_24_]^4–^.^20^ Cluster **3** was also characterized by ESI-MS analysis (see the Experimental Section and Supporting Information).

The last method we exploited to synthesize new nanoclusters involved the decomposition of a cluster precursor, in this case [Rh_12_Sb(CO)_27_]^3–^, and the subsequent condensation of the obtained fragments. Indeed, the cluster disaggregation may occur via thermolysis^22^ or by use of a coordinative ligand to remove metal atoms.^32^ Therefore, we directly reacted the icosahedral [Rh_12_Sb(CO)_27_]^3–^ with PPh_3_, in acetonitrile and under N_2_ atmosphere, with the aim of subtracting Rh atoms from the parent species by forming Rh–PPh_3_ complexes. We stopped the addition of the phosphine after 0.8 equiv, when the characteristic IR peaks of the starting cluster disappeared and were replaced by those of a new, unknown species. Some di-isopropyl ether was directly layered onto the mother solution, without performing any workup, and a few crystals suitable for a structural characterization were obtained. However, the resulting product was a lower nuclearity species than the starting cluster, as PPh_3_ partly broke the icosahedral metal skeleton but stabilized the new compound by acting as a ligand, replacing some COs. Indeed, the new cluster was identified as [Rh_10_Sb(CO)_21_PPh_3_]^3–^ (**4**), in the form of its [NEt_4_]^+^ salt.

Thanks to the presence of the phosphine ligand onto the metal skeleton, cluster **4** was also characterized by NMR spectroscopy. The ^31^P NMR spectrum registered in CD_3_CN at 298 K shows a doublet of multiplets centered at δ_P_ 33.38 ppm, with ^1^*J*_Rh–P_ = 249 Hz and ^2^*J*_Rh–P_ = 5 Hz. In particular, the presence of the ^2^*J*_Rh–P_ indicates that the PPh_3_ is coordinated to a proper cluster, as opposed to a metal complex. Indeed, P couples not only with the Rh atom to which is bound but also with some of the others that constitute the skeleton. Its low value is due to the delocalized electronic density within the Rh–Rh interactions. The coupling values are consistent with those reported for other Rh clusters coordinated to triphenylphosphines.^33^

### Molecular Structures of the [Rh_20_Sb_3_(CO)_36_]^3–^, [Rh_28–*x*_Sb_*x*_(CO)_44_]^6–^, [Rh_21_Sb_2_(CO)_38_]^5–^, and [Rh_10_Sb(CO)_21_PPh_3_]^3–^ Anionic Clusters

All presented clusters have been structurally characterized by single-crystal X-ray diffraction, and only for cluster **2** are the crystal data not of sufficient quality for its unambiguous formulation. Crystallographic details are reported in Table 1, while the most relevant bond lengths are provided in the Supporting Information. In the solid state, all compounds are arranged in an ionic fashion, so the anionic clusters are surrounded by the cations. The solvent molecules, where present, fill the voids to maximize the packing density. No significant intermolecular hydrogen bonds have been found.

**Table 1:** Crystallographic Data for Clusters **1**, **3**, and **4**

compound	**1**[NEt_4_]_3_·2(CH_3_)_2_CO	**3**[NEt_4_]_5_·4CH_3_CN	**4**[NEt_4_]_3_·CH_3_CN
formula	C_66_H_72_N_3_O_38_Rh_20_Sb_3_	C_86_H_112_N_9_O_38_Rh_21_Sb_2_	C_67_H_82_N_4_O_21_PRh_10_Sb
*F*_w_	3898.71	4284.45	2461.18
crystal system	triclinic	triclinic	monoclinic
space group	*P*1̅	*P*1̅	*P*_2_1/*c*
*a* (Å)	14.732(3)	14.705(7)	21.9963(11)
*b* (Å)	15.089(5)	17.825(8)	16.6173(8)
*c* (Å)	24.650(8)	22.389(9)	21.1485(10)
α (deg)	100.98(4)	94.705(10)	90
β (deg)	96.80(2)	94.827(8)	91.3080(10)
γ (deg)	117.19(2)	92.555(8)	90
cell volume (Å^3^)	4650(2)	5820(4)	7728.2(6)
*Z*	2	2	4
*D* (g/cm^3^)	2.813	2.445	2.115
μ (mm^–1^)	4.376	3.419	2.511
*F* (000)	3692	4084	4784
θ limits (deg)	1.577–24.999	1.392–25.000	1.536–25.000
index ranges	–17 ≤ *h* ≤ 17	–17 ≤ *h* ≤ 17	–26 ≤ *h* ≤ 26
	–17 ≤ *k* ≤ 17	–21 ≤ *k* ≤ 21	–19 ≤ *k* ≤ 19
	–29 ≤ *l* ≤ 29	–26 ≤ *l* ≤ 26	–25 ≤ *l* ≤ 25
reflections collected	59360	70274	87637
independent reflections	16330	20488	13515
	[*R*(int) = 0.2209]	[*R*(int) = 0.0353]	[*R*(int) = 0.0248]
completeness to θ_max_	99.7%	99.8%	99.4%
data/restraints/parameters	16330/632/1271	20484/293/1514	13515/310/1010
goodness of fit	0.969	1.019	1.148
*R*_1_ (*I* > 2σ(*I*))	0.0708	0.0321	0.0233
*wR*_2_ (all data)	0.1908	0.0816	0.0538
largest diff. peak and hole, e Å^–3^	1.543 and −1.520	2.477 and −1.305	1.059 and −1.256

**Figure 1:**
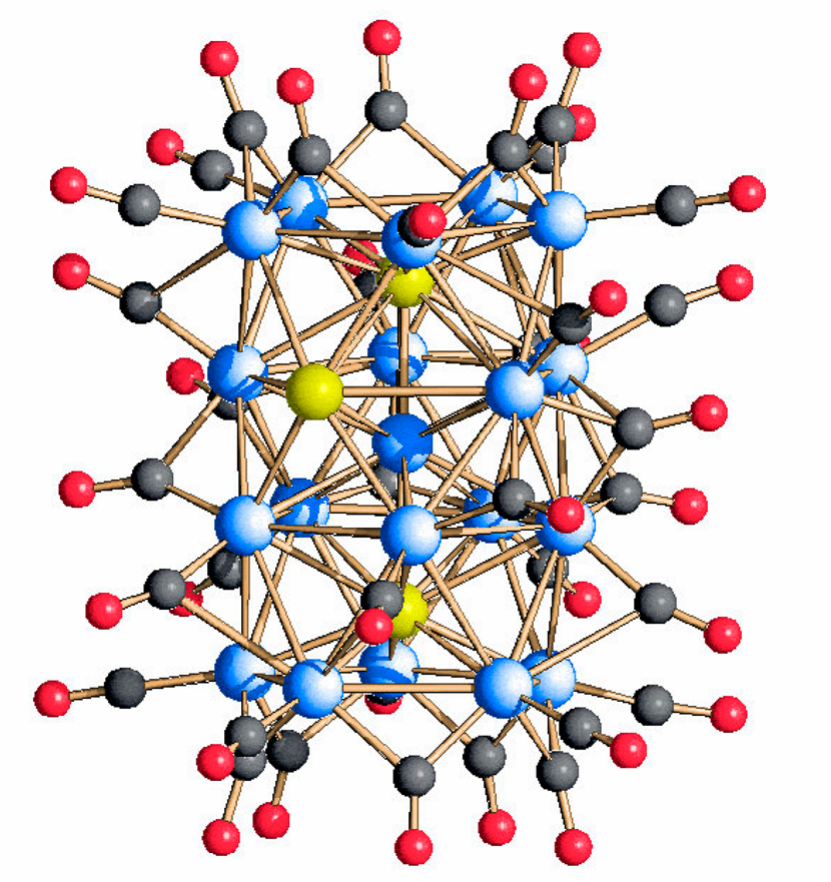
Molecular structure of [Rh_20_Sb_3_(CO)_36_]^3–^, **1**. Color key: Rh, blue; Sb, yellow; C, gray; and O, red.

**Figure 2:**
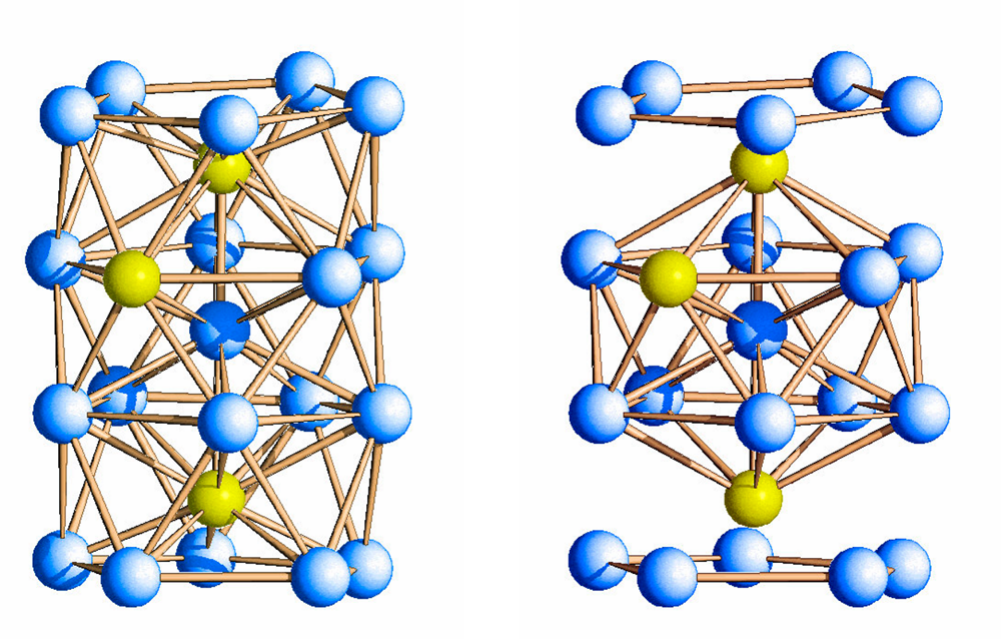
Metal skeleton of [Rh_20_Sb_3_(CO)_36_]^3–^ (left), and its breakdown into a Rh-centered Rh_9_Sb_3_ icosahedron with the two opposite Sb vertexes capped by pentagonal Rh_5_ faces (right). Rh is depicted in blue, and Sb is in yellow.

The molecular structure of [Rh_20_Sb_3_(CO)_36_]^3–^(**1**) is represented in Figure 1. Its metal skeleton (Figure 2 and the Supporting Information) consists of a Rh-centered Rh_9_Sb_3_ icosahedron with the two opposite Sb vertexes capped by two pentagonal Rh_5_ faces, and it is stabilized by 36 carbonyl ligands, of which 19 are terminally bonded, 15 are edge-bridging, and 2 are face-bridging. The Rh–Rh distances present an average value of 2.892(23) Å and are overall longer than the Rh–Sb ones. More specifically, the Rh–Sb bond lengths involving the interstitial Sb atoms (Sb(1) and Sb(3)) with the inner Rh(6) are the shortest (2.553(4) Å), whereas those with the peripheral Rh atoms have an average value of 2.800(13) Å. Conversely, the Rh–Sb bond lengths involving the surface Sb atom show a mean value of 2.837(8) Å, close to the Rh–Rh bond contacts. These distances are in line, albeit slightly shorter, with those observed in the icosahedral [Rh_12_Sb(CO)_27_]^3–^ species. Finally, the unique Sb–Sb bond distance is 3.014(3) Å, significantly longer than that in the elementary Sb (2.84 Å).

The maximum length and width of **1**, assessed from the outermost oxygen atoms of the CO ligands and considering twice the van der Waals oxygen radius, are 1.50 and 1.00 nm, placing this compound in the nanometer regime.

**Figure 3:**
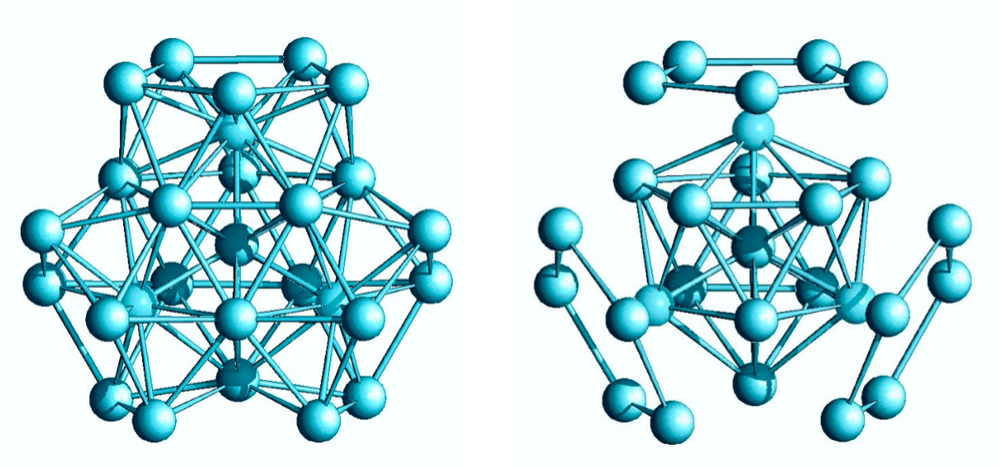
Metal skeleton of [Rh_28–*x*_Sb_*x*_(CO)_44_]^6–^, **2**, (left), and its breakdown into a centered [RhSb]_12_ icosahedron with three vertexes capped by pentagonal faces (right).

The metal skeleton of [Rh_28–*x*_Sb_*x*_(CO)_44_]^6–^ (**2**) is depicted in Figure 3, and it can be described as the fusion of three uncompleted centered [RhSb]_11_ icosahedra sharing one vertex, represented by the inner metal atom in the whole cluster. It may be also described as a centered [RhSb]_12_ icosahedron where the three vertexes are capped each by a pentagonal face. Considering the available structural data, it is not possible to indisputably identify the interstitial atoms, while there is no doubt that the external ones, coordinated to the CO ligands, are rhodium. So far, this species represents the larger RhSb carbonyl cluster to date.

**Figure 4:**
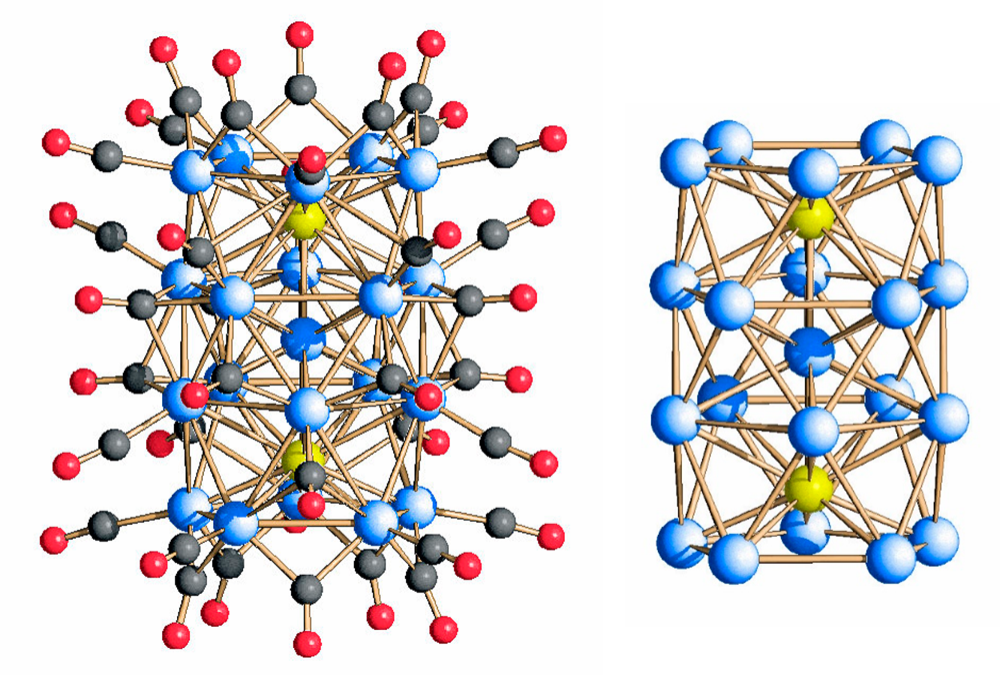
Molecular structure of [Rh_21_Sb_2_(CO)_38_]^5–^, **3** (left), and its metal skeleton (right). Color key: Rh, blue; Sb, yellow; C, gray; and O, red.

The molecular structure of [Rh_21_Sb_2_(CO)_38_]^5–^ (**3**) is shown in Figure 4. The metal framework is stabilized by 38 CO ligands, two more than **1** because of the additional Rh atom, of which 20 are terminally bonded, 14 are edge-bridging, and the remaining 4 are face-bridging. The metal skeleton of [Rh_21_Sb_2_(CO)_38_]^5–^ is nearly identical to that of [Rh_20_Sb_3_(CO)_36_]^3–^, with the sole difference that a Sb atom in the latter is replaced by a Rh atom in the former. Therefore, the metal framework can be described by a Rh-centered Rh_10_Sb_2_ icosahedron whose opposite Sb vertexes are capped by two pentagonal Rh_5_ faces. In terms of interatomic distances, the two halved cluster molecules in the independent unit are only marginally different. The Rh–Rh bond lengths in the first and second isomer present average values of 2.8741(80) and 2.8723(83) Å, respectively. These are both slightly shorter than the average length of the Rh–Rh bonds in **1** (2.892(23) Å). The Rh–Sb bond distances involving the inner Rh atoms (Sb(1)–Rh(2) in the first isomer and Sb(21)–Rh(22) in the second one (see the Supporting Information for labels)) are the shortest if compared with those involving the peripheral Rh atoms, 2.5128(9) and 2.5136(9) Å, respectively. They are also shorter than the corresponding bonds in cluster **1** (2.553(4) Å). The bond distances with the outer Rh atoms are significantly longer, with average values of 2.8242(60) and 2.8212(43) Å in the two isomers, and they are also slightly longer than those in cluster **1** (average 2.800(13) Å).

The maximum length of **3** between the outermost oxygen atoms of the carbonyl ligands, and including twice the oxygen van der Waals radius, is 1.50 nm for both isomers, while the width measures 1.00 nm. As expected, its size matches that of cluster **1**.

**Figure 5:**
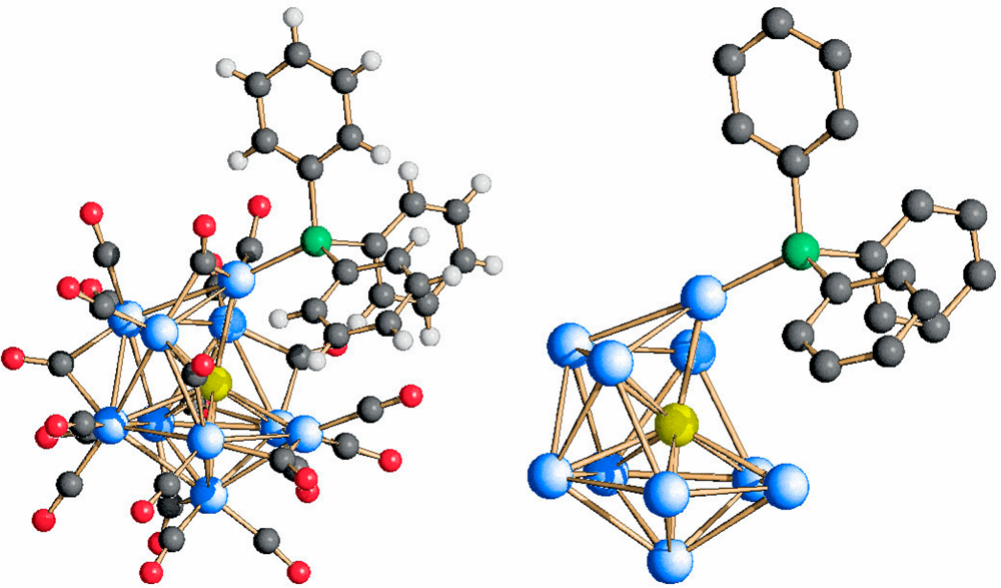
Molecular structure of [Rh_10_Sb(CO)_21_PPh_3_]^3–^, **4** (left), and its metal skeleton (right). Color key: Rh, blue; Sb, yellow; P, green; C, gray; O, red; and H, white.

The molecular structure of [Rh_10_Sb(CO)_21_PPh_3_]^3–^ (**4**) is illustrated in Figure 5. Its metal skeleton is based on a broken icosahedron made of 10 Rh atoms, centered by the unique Sb atom and coordinated to 1 PPh_3_, as well as to 21 carbonyl ligands, among which 13 are terminally bonded to the Rh atoms and the remaining 8 are edge-bridged. The Sb–Rh bond lengths present an average value of 2.7265(9) Å, whereas the Rh–Rh distances are longer, with an average of 2.9315(46) Å. Due to the partially open icosahedral cage, these values are lower than the corresponding ones observed in both [Rh_12_Sb(CO)_24_]^4–^ and [Rh_12_Sb(CO)_27_]^3–^.

The maximum size of **4** is 1.50 nm, measured from the outermost oxygen atoms of the carbonyl ligands to the outermost hydrogen atoms of the phosphine ligand and including the oxygen van der Waals radius and the hydrogen one, like cluster **1** and **3**. However, if the PPh_3_ ligand is ignored for homogeneity with the other presented species, then the cluster size decreases down to 1.30 nm, slightly smaller than the integer [Rh_12_Sb(CO)_27_]^3–^ parent compound (1.40 nm).

### Electron Counting

In the field of deltahedral clusters, the model for their electron counting comes from the borane chemistry, according to the Polyhedral Skeleton Electron Pair Theory (PSEPT) by Wade and Mingos.^34,35^ In a *closo* borane with *N* number of atoms, the valence molecular orbitals (MOs) are [*N* + (*N* + 1)], of which *N* are used for the localized B–H bonds, and the remaining (*N* + 1) MOs to hold the metal cage. In case of compounds involving transition metal atoms, the additional five d orbitals must be taken into account; therefore, the original counting develops into the alternative [5*N* + *N* + (*N* + 1) = 7*N* + 1] CVMOs (Cluster Valence Molecular Orbitals). When condensed polyhedral clusters are involved, their electron counting can be better derived from the fusion of smaller regular polyhedra through vertexes, edges, or faces, whose electrons should be taken out from the counting. For instance, [Rh_20_Sb_3_(CO)_36_]^3–^ should have 134 MOs, or 268 CVEs (Cluster Valence Electrons), because its structure can be seen as three pentagonal antiprisms (3 × 146 CVEs) fused through two pentagonal faces (−2 × 80 CVEs), with the external Sb shared by two of the pentagonal antiprisms (−2 × 5 CVEs). The CVEs for [Rh_20_Sb_3_(CO)_36_]^3–^ are indeed 268, given by the 9 × 20 rhodium atoms (180), the 2 × 36 carbonyl ligands (72), the 5 × 2 interstitial Sb atoms (10), the surface Sb atom (3), and the negative charge (3); this Rh–Sb cluster, therefore, is perfectly in line with the above electron counting rule.

As for [Rh_21_Sb_2_(CO)_38_]^5–^, since the structure consists of three pentagonal antiprisms (3 × 146 CVEs) fused through pentagonal faces (−2 × 80 CVEs), it should have 278 CVEs. Actually, [Rh_21_Sb_2_(CO)_38_]^5–^ presents 280 CVEs, given by the 9 × 21 rhodium atoms (189), the 2 × 38 carbonyl ligands (76), the 5 × 2 Sb atoms (10), and the negative charge (5). This species slightly deviates from the PSEPT; nevertheless, the theory has proved to be not always appropriate to predict the CVEs number as the cluster nuclearity increases.^36^

Ultimately, [Rh_10_Sb(CO)_21_PPh_3_]^3–^ should present 142 CVEs, since its structure can be seen as a *closo*-bicapped square antiprism exhibiting 7*N* + 1 MOs, or 14*N* + 2 CVEs. As a matter of fact, this compound shows 142 CVEs, given by the 9 × 10 rhodium atoms (90), the 2 × 21 carbonyl ligands (42), the 5 × 1 Sb atom (5), and the negative charge (3), proving to conform to the PSEPT.

### Electrochemical and Spectroelectrochemical Studies of [Rh_21_Sb_2_(CO)_38_]^5–^ (**3**)

The electrochemical properties of transition-metal carbonyl clusters have been investigated more and more over the past decades thanks to the increasing number of available isolated and structurally characterized compounds. The variety of composition, structure, and nuclearity of such compounds, however, has hindered systematic studies of their properties in relationship with the above characteristics. Nonetheless, it has been experimentally demonstrated that clusters may show interesting electronic properties, for instance multivalent behaviors, when they possess some ad hoc features that enhance their stability under redox conditions.^37^ The presence of heteroatoms reinforcing the metal skeleton is one of them, as in the case of the cationic [Au_24_Pd(PPh_3_)_10_(SC_2_H_4_Ph)_5_Cl_2_]^+^ heteroleptic compound^38^ or in the [H_6–*n*_Ni_31_P_4_(CO)_39_]^*n*−^ (*n* = 4 and 5) and [Ni_32_C_6_(CO)_36_]^*n*−^ (*n* = 5–10) homoleptic carbonyl species.^39,40^ The latter two clusters present the additional synergy arising from the shielding of the carbonyl shell and the presence of interstitial metal atoms.

We studied the [Rh_21_Sb_2_(CO)_38_]^5–^ species (**3**) by CV and *in situ* infrared spectroelectrochemistry (IR SEC), as it could be obtained in high yields. Its redox properties were first examined by CV in CH_3_CN/[N^*n*^Bu_4_][PF_6_] solution, at different scan rates, namely 5, 20, 50, 100, 200, and 400 mV/s. The voltammetric profile between −0.4 and −2.0 V (Figure S8) registered at 0.2 V s^–1^ shows several reduction steps, but the low currents and resolution did not allow to derive the potentials and their reversibility degree. Such CV profiles, characterized by very low current intensity, are not uncommon in high-nuclearity clusters.^39^ A more intense oxidation process is visible between −0.4 and 0.0 V, and it appears electrochemically quasi-reversible (Δ*E*_p_ = 150 mV) and chemically reversible within the cyclic voltammetric time scale. The redox chemistry of **3** was also studied by IR SEC in an OTTLE cell.^41^ When the potential of the working electrode was swept between +0.6 and −1.9 V vs Ag pseudoreference electrode, the ν_CO_ bands of the cluster shifted toward higher (or lower) wavenumbers upon each anodic (or cathodic) step, with differences within a range of 14–20 cm^–1^. These shifts appear to be consistent with monoelectronic steps, as previously observed, for instance, in high-nuclearity platinum and rhodium carbonyl clusters.^42,43^ This assumption is in line with chemical reduction and oxidation experiments (see the following sections), although the low current in the CV analysis did not allow a direct determination of the number of the exchanged electrons.

**Figure 6:**
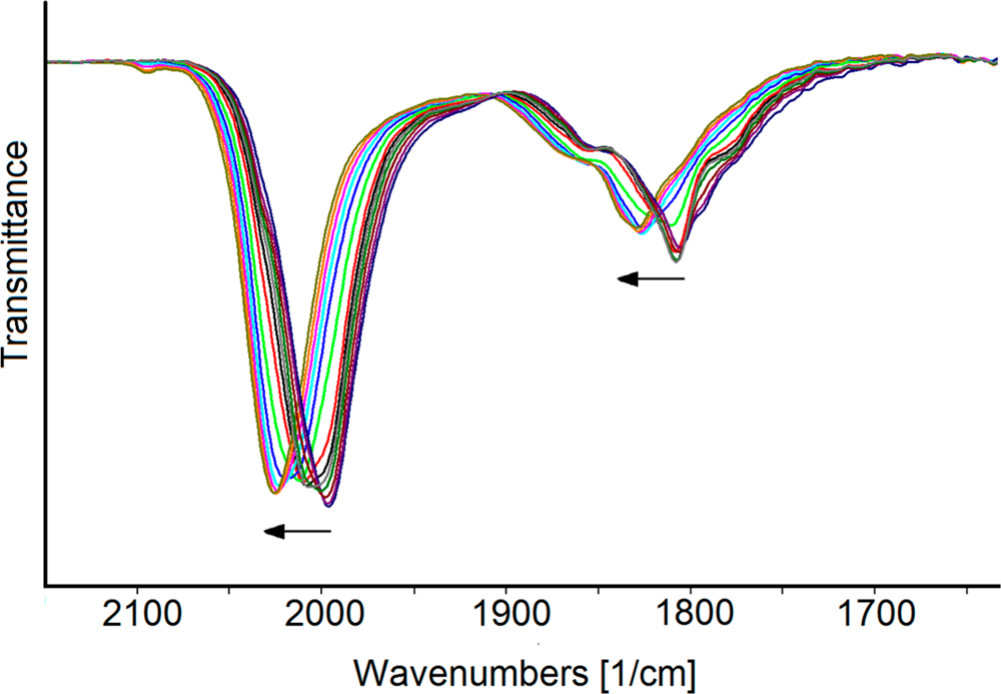
IR spectral changes of a CH_3_CN solution of **3** recorded in an OTTLE cell during the progressive increase of the potential from −0.6 to +0.6 V vs Ag pseudoreference electrode (scan rate 1 mV s^–1^), with [N^*n*^Bu_4_][PF_6_] (0.1 mol dm^–3^) as supporting electrolyte. The absorptions of the solvent and the supporting electrolyte have been subtracted.

Figure 6 shows the infrared spectroelectrochemical sequence recorded during the progressive oxidation of **3**, which occurred between −0.6 and +0.6 V. Under these conditions, a progressive shift of both the terminal and edge-bridging carbonyl bands from 1996 and 1806 cm^–1^ to 2026 and 1829 cm^–1^, respectively, was observed. The chemical reversibility of this oxidation was verified through a backward potential, which restored the original IR spectrum.

**Figure 7:**
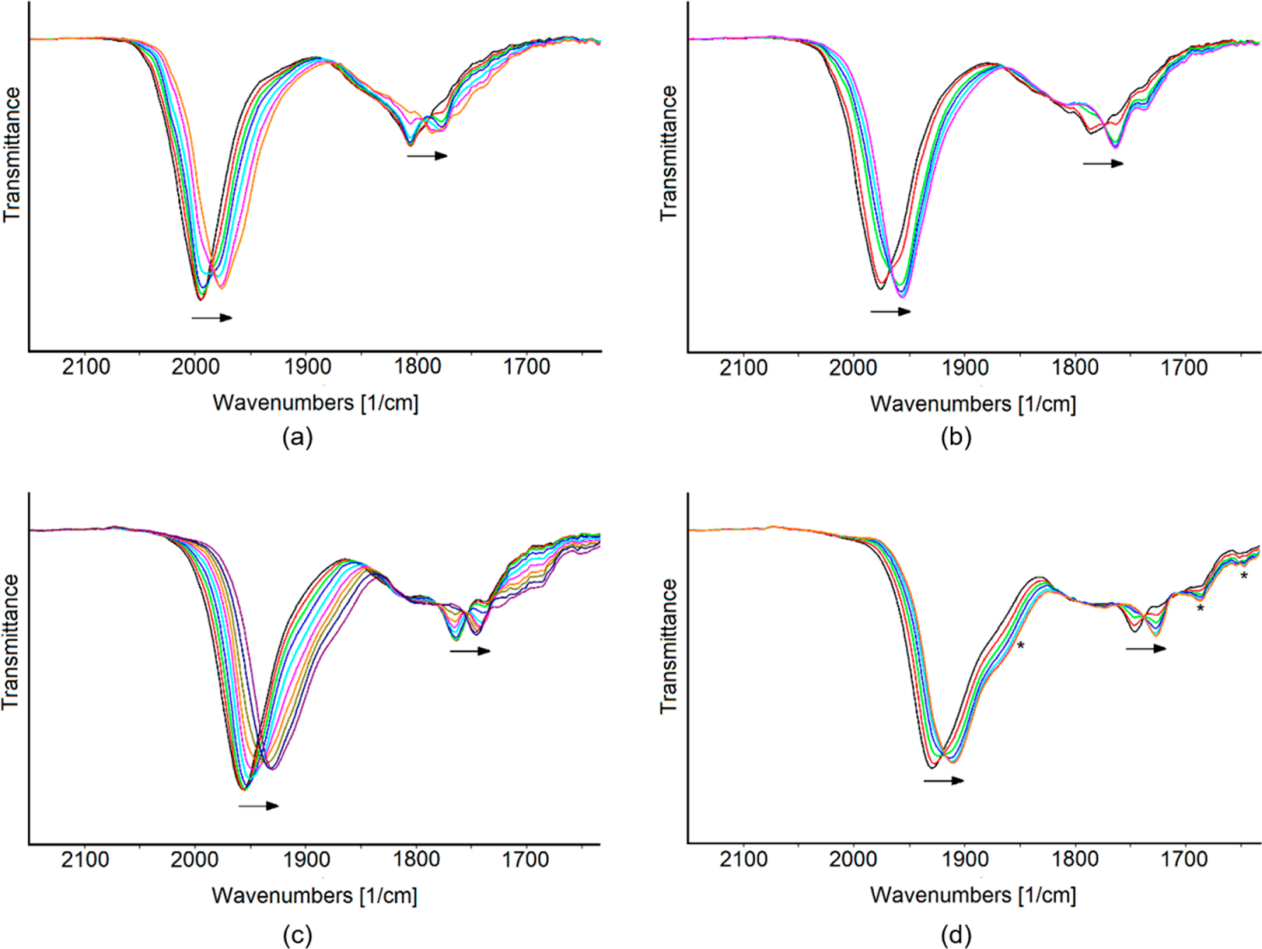
IR spectral changes of a CH_3_CN solution of **3** recorded in an OTTLE cell during the progressive decrease of the potential (a) from −0.6 to −0.96 Vm (b) from −0.96 to −1.32 Vm (c) from −1.32 to −1.90 V, and (d) from −1.90 to −2.1 V vs Ag pseudoreference electrode (scan rate 1 mV s^–1^). The signals marked with an asterisk in (d) are at 1851, 1684, and 1636 cm^–1^.

The IR SEC sequence was also recorded upon the stepwise reduction of **3** (Figure 7), and between −0.6 and −1.9 V, three processes were evident. We verified that no decomposition of the electro-generated species occurred because the starting IR spectrum of **3** was regenerated when the potential returned to the initial value. A further progressive shift at lower wavenumbers (1911 cm^–1^ for the terminal COs, Figure 7d) was observed on decreasing the potential down to −2.1 V. In this case, the reverse oxidation backscan did not completely restore the original IR spectrum, and weak bands (asterisked peaks in Figures S9 and 7d) pointed out a relatively slow decomposition of this more reduced species.

In the oxidation and reduction sequences (Figure 7c), the carbonyl absorptions shifted to higher or lower frequencies, respectively, without a well-defined isosbestic point. This may indicate the presence of more than two compounds. This phenomenon has already been found for other high-nuclearity clusters,^39,44^ and the coexistence of more than two species in one IR spectrum under electrochemical investigation is not uncommon in transition metal clusters. For instance, spectroelectrochemical studies on the [Pt_38_(CO)_44_]^2–^ cluster showed that “the passage [Pt_38_(CO)_44_]^2–/3–^ does not give rise to an isosbestic point because of the fast set up of the [Pt_38_(CO)_44_]^3–^ ⇄ [Pt_38_(CO)_44_]^4–^ equilibrium”.^45^ In light of all the above and because of the chemical reversibility of the whole process, we attributed these additional species to transient oxidation states of cluster **3**, and not to isomerism equilibria or decomposition products. Our hypothesis is in agreement with the results of spectral deconvolutions performed on some selected IR sequences registered during both oxidation and reduction processes, which allowed us to determine their single absorbance contributions. A detailed description of this analysis is reported in the Supporting Information.

We also performed a chemical oxidation and reduction of cluster **3** through a stepwise addition of tropylium tetrafluoborate in CH_3_CN solution or of Na/naphthalene in dimethylformamide (DMF), respectively. The resulted IR spectra matched those observed through the IR SEC analyses, although it was not possible to reach the more reduced species. This misalliance between chemical and electrochemical redox states may occur in carbonyl clusters, as the more negatively (or positively) charged species are often stable only on the time scale of electrochemical experiments.^46^

**Figure 8:**
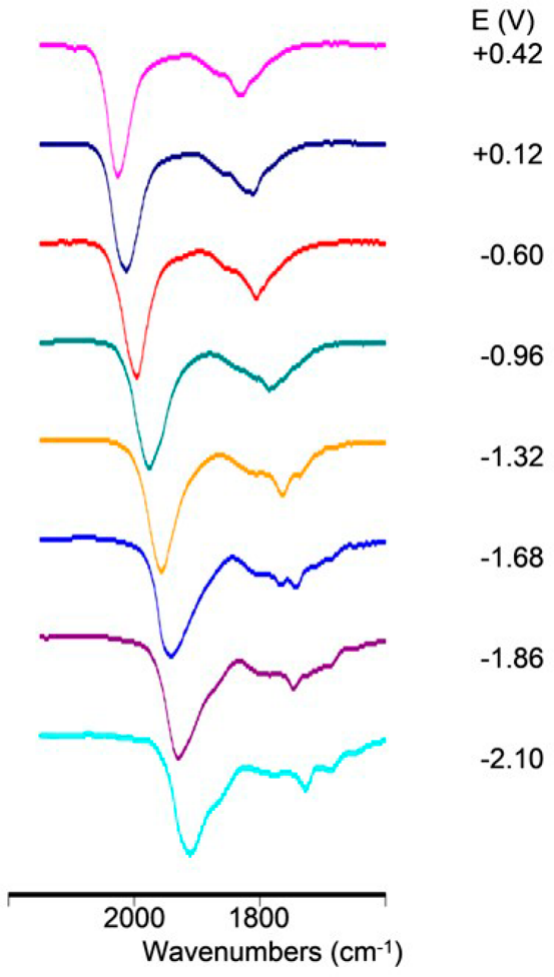
Selected IR spectra of [Rh_21_Sb_2_(CO)_38_]^*n–*^ as a function of the potential *E* (vs Ag pseudoreference electrode). The initial spectrum (*n* = 5) is at −0.60 V.

In spite of the poor results of the electrochemistry, the combined study of *in situ* IR SEC of [Rh_21_Sb_2_(CO)_38_]^5–^, the peak fitting analyses through spectral deconvolution and the chemical redox experiments allowed us to suggest that cluster **3** is a multivalent species with a rich redox chemistry that can stably exist in several oxidation states. As for their number and labeling, the experimental data at our disposal do not allow an indisputable assignment. Figure 8 shows a summary of the obtained IR spectra ad different potentials.

## Experimental Section

All reactions and compounds were handled using the standard Schlenk technique and under either nitrogen or carbon monoxide atmosphere. Solvents were dried and degassed before use, THF was dehydrated with Na-benzophenone and distilled under nitrogen. Ammonium salts and SbCl_3_ reagents were commercial products. The [Rh_7_(CO)_16_]^3–^ cluster precursor was prepared according to literature.^31^ IR spectra were recorded on a PerkinElmer Spectrum One interferometer in CaF_2_ cells.

EDS experiments were performed on a SEM Zeiss EVO 50 equipped with EDS Detector Oxford Model INCA 350 working at 20 kV of acceleration energy. Positive/negative-ion mass spectra were recorded in CH_3_CN solutions on a Waters Micromass ZQ 4000 by using electrospray (ES) ionization. Experimental conditions: 2.56 kV ES-probe voltage, 10 V cone potential, 250 L h^–1^ flow of N_2_ spray-gas, incoming-solution flow 20 μL min^–1^. ^31^P NMR measurements were performed on a Varian Mercury Plus 400 MHz instrument. The phosphorus chemical shifts were referenced to external H_3_PO_4_ (85% in D_2_O).

Single-crystal X-ray diffraction experiments were performed at 100 K on a Bruker Apex II diffractometer, equipped with a CCD (in the case of **1**, **3**, and **4**) or a CMOS (in the case of **2**) detector, by using Mo Kα radiation. Data were corrected for Lorentz polarization and absorption effects (empirical absorption correction SADABS).^47^ Structures were solved by direct methods and refined by full-matrix least-squares based on all data using *F*^2^.^48^ Hydrogen atoms were fixed at calculated positions and refined by a riding model. All non-hydrogen atoms were refined with anisotropic displacement parameters, including disordered atoms. Structure drawings were made with SCHAKAL99.^49^ In the structural models of clusters **1** and **3**, some cations and/or solvent molecules were treated as positionally disordered. More specifically, in cluster **1**, one cation was split in two positions, using the necessary anisotropic displacement parameter restraints, and their relative occupancy factor resulted to be close to 50% each. For clusters **3** and **4**, in addition to the same type of disorder in one cation (**3** and **4**) and one acetonitrile molecule (**3**), we found some more severe disorder involving the solvent molecules; therefore, we applied the PLATON SQUEEZE tool.^50^

Electrochemical measurements were performed with a PalmSens4 instrument interfaced to a computer employing PSTrace5 electrochemical software. CV measurements were carried out at room temperature under Ar in CH_3_CN solutions containing [N^*n*^Bu_4_][PF_6_] (0.1 mol dm^–3^) as the supporting electrolyte. HPLC-grade CH_3_CN (Sigma-Aldrich) was stored under argon over 3 Å molecular sieves. Electrochemical-grade [N^*n*^Bu_4_][PF_6_] was purchased from Fluka and used without further purification. Cyclic voltammetry was performed in a three-electrode cell; the working and the counter electrodes consisted of a Pt disk and a Pt gauze, respectively, both sealed in a glass tube. An Ag/AgCl, KCl saturated electrode mounted with a salt bridge containing the CH_3_CN/[N^*n*^Bu_4_][PF_6_] electrolyte and separated by a Vycor frit was employed as a reference electrode. The three-electrode lab-built cell was predried by heating under vacuum and filled with argon. The Schlenk type construction of the cell maintained anhydrous and anaerobic conditions. The solution of supporting electrolyte, prepared under argon, was introduced into the cell, and the CV of the solvent was recorded. The analyte was then introduced and voltammograms were recorded. Under the present experimental conditions, the one-electron oxidation of ferrocene occurs at *E*° = +0.42 V vs Ag/AgCl. Infrared spectroelectrochemical measurements were carried out using an optically transparent thin-layer electrochemical (OTTLE) cell^39^ equipped with CaF_2_ windows, platinum mini-grid working and auxiliary electrodes, and silver wire pseudoreference electrode. During the microelectrolysis procedures, the electrode potential was controlled by a PalmSens4 instrument interfaced to a computer employing PSTrace5 electrochemical software. We used argon-saturated CH_3_CN solutions of the compound under study, containing [N^*n*^Bu_4_][PF_6_] 0.1 M as the supporting electrolyte. The *in situ* spectroelectrochemical experiments have been performed by collecting spectra of the solution at constant time intervals during the oxidation or reduction, obtained by continuously increasing or lowering the initial working potential at a scan rate of 1.0 mV/s. IR spectra were recorded on a PerkinElmer Spectrum 100 FT-IR spectrophotometer.

### Synthesis of [Rh_20_Sb_3_(CO)_36_]^3–^

An acetonitrile solution of SbCl_3_ (0.118 g, 0.52 mmol) was slowly added to a solution of [Rh_7_(CO)_16_][NEt_4_]_3_ (0.700 g, 0.45 mmol) in the same solvent, under CO atmosphere, and in a 1.15:1 molar ratio, respectively. After 4 h, the resulting brown solution was dried under vacuum, and the solid was washed with water (150 mL), ethanol (150 mL), and THF (50 mL). [Rh_20_Sb_3_(CO)_36_]^3–^ was extracted in acetone (30 mL), and black crystals of [Rh_20_Sb_3_(CO)_36_][NEt_4_]_3_·2(CH_3_)_2_CO (yield ≈ 60% based on Rh) were obtained by layering *n*-hexane on the solution. [Rh_20_Sb_3_(CO)_36_][NEt_4_]_3_ is soluble in acetone, acetonitrile, and DMF and stable, but not soluble, in water. Its IR spectrum recorded in CH_3_CN shows ν_CO_ absorptions at 2030 (vs) and 1833 (ms) cm^–1^. ESI-MS spectrum of [Rh_20_Sb_3_(CO)_36_][NEt_4_]_3_ displays many groups of peaks because of its instability in the experimental conditions. The only groups of peaks attributable to the integer species start at 1782 and 1135 *m*/*z* ({[Rh_20_Sb_3_(CO)_36–35_][NEt_4_]}^2–^ and [Rh_20_Sb_3_(CO)_35–32_]^3–^, respectively). The others are due to the breaking of the metal skeleton into species such as [Rh_10_Sb_3_(CO)_18_]^2–^.

### Synthesis of [Rh_28–*x*_Sb_*x*_(CO)_44_]^6–^

An acetonitrile solution of SbCl_3_ (0.145 g, 0.64 mmol) was slowly added to a solution of [Rh_7_(CO)_16_][NEt_4_]_3_ (1.420 g, 0.91 mmol) in the same solvent, under N_2_ atmosphere, and in a 0.70:1 molar ratio, respectively. After 3 h, the resulting brown solution was dried under vacuum, and the solid was washed with water (150 mL), ethanol (100 mL), and THF (40 mL). [Rh_28–*x*_Sb_*x*_(CO)_44_]^6–^ was extracted in acetone (30 mL) with impurities of [Rh_21_Sb_2_(CO)_38_]^5–^, and by layering *n*-hexane on the solution we obtained black crystals of [Rh_28–*x*_Sb_*x*_(CO)_44_][NEt_4_]_6_ (very low yield). Its IR spectrum recorded in CH_3_CN shows ν_CO_ absorptions at 1996 (vs), 1857 (w), 1806 (m), and 1773 (w) cm^–1^. ESI-MS analysis on [Rh_28–*x*_Sb_*x*_(CO)_44_][NMe_4_]_6_ show signals at 1439, 1415, and 1371 *m*/*z* that we assigned to the {[Rh_25_Sb_3_(CO)_44_][NMe_4_]_2_}^3–^, {[Rh_25_Sb_3_(CO)_44_][NMe_4_]}^3–^, and {[Rh_25_Sb_3_(CO)_42_]}^3–^ ions, respectively. EDS experiments showed an atomic composition of Rh and Sb in the cluster equal to 91.8 and 8.2% (±0.3%).

### Synthesis of [Rh_21_Sb_2_(CO)_38_]^5–^

An acetone solution of SbCl_3_ (0.059 g, 0.257 mmol) was slowly added to a solution of [Rh_7_(CO)_16_][NEt_4_]_3_ (0.500 g, 0.321 mmol) in the same solvent, under N_2_ atmosphere, and in a 0.80:1 molar ratio, respectively. After 3 h, the insoluble residue that had precipitated during the reaction was dried in vacuum and extracted in acetonitrile (20 mL), and black crystals of [Rh_21_Sb_2_(CO)_38_][NEt_4_]_5_·4CH_3_CN (yield ≈ 45% based on Rh) were obtained by layering di-isopropyl ether on the solution. [Rh_21_Sb_2_(CO)_38_][NEt_4_]_5_ is soluble in acetonitrile and DMF and stable, but not soluble, in water. Its IR spectrum recorded in CH_3_CN shows ν_CO_ absorptions at 1995 (vs) and 1805 (m) cm^–1^. ESI-MS spectrum mainly exhibits two groups of signals starting at 1101 and 1716m/z. The first group is attributable to the [Rh_21_Sb_2_(CO)_32_]^3–^ species, while the second one is related to the {[Rh_21_Sb_2_(CO)_32_][NEt_4_]}^2–^ ion. Both are accompanied by further peaks, due to consecutive CO losses.

### Synthesis of [Rh_10_Sb(CO)_21_PPh_3_]^3–^

A sample of [Rh_12_Sb(CO)_27_][NEt_4_]_3_ (0.260 g, 0.104 mmol) was dissolved in acetonitrile, and a second solution of PPh_3_ (0.022 g, 0.083 mmol) in the same solvent was slowly added to the former, under N_2_, in a 1:0.8 molar ratio. After a few hours, the resulting mother solution was filtered, and di-isopropyl ether was layered on top, allowing us to obtain [Rh_10_Sb(CO)_21_PPh_3_][NEt_4_]_3_·CH_3_CN in a crystalline form (yield ≈ 30% based on Rh). The compound is soluble in acetone, acetonitrile, and DMF. Its IR spectrum presents ν_CO_ in CH_3_CN: 1991 (vs), 1981(sh), 1844(m), 1805(m), and 1762(ms) cm^–1^. ^31^P NMR (CD_3_CN, 298 K) δ_P_(ppm): 33.38 (m, ^1^*J*_Rh–P_ = 249 Hz and ^2^*J*_Rh–P_ = 5 Hz).

## Conclusions

In this paper, we present the synthesis and characterization of three large and atomically precise Rh–Sb carbonyl nanoclusters, which we obtained by reacting the [Rh_7_(CO)_16_]^3–^ cluster precursor with Sb^3+^ at different reaction conditions, exploiting their redox condensation process. More specifically, the [Rh_20_Sb_3_(CO)_36_]^3–^ trianion was obtained under CO atmosphere and in acetonitrile, while the larger [Rh_28–*x*_Sb_*x*_(CO)_44_]^6–^ was isolated by working under N_2_. Conversely, the [Rh_21_Sb_2_(CO)_38_]^5–^ species was still synthesized under N_2_ but using the less solubilizing acetone as solvent. All the aforementioned products were separated from the final reaction mixtures by subsequent extractions with solvents at increasing polarity. These results confirmed the effectiveness of the condensation–reaction method to prepare not only small heterometallic clusters but also metal nanoparticles that can be still characterized at a molecular level.

We applied another known method, which involves the decomposition of a cluster precursor and the subsequent condensation of the obtained unstable fragments, to assess whether it could be suitable to the Rh–Sb system. In this case, we prepared the heterometallic [Rh_12_Sb(CO)_27_]^3–^ cluster and reacted it with PPh_3_. However, the latter stabilized the fragmented compound by acting as a ligand; therefore, we isolated the lower-nuclearity [Rh_10_Sb(CO)_21_PPh_3_]^3–^ heteroleptic cluster. In spite of being much smaller than the other species presented in this work, its dimensions still belong to the nanometer regime.

In the case of **2**, EDS analysis was also performed, while **4** was characterized by ^31^P NMR spectroscopy thanks to the presence of the phosphine ligand. Clusters **1**, **2**, and **3** were additionally characterized by ESI-MS spectrometry, which confirmed the sufficient robustness of the Rh–Sb carbonyl clusters in the experimental conditions even with this kind of nuclearity, as opposed to, for instance, Ni-containing species of similar size.^51^

All clusters have been characterized by IR spectroscopy, and their molecular structures completely determined by single-crystal X-ray diffraction. Notably, these Rh–Sb compounds show a distinct propensity to adopt icosahedral-based geometries, similarly to what observed for Au,^52,53^ Ag,^54^ and Pd^55−57^ clusters. Despite the fact that they do not strictly represent a prototypic arrangement of the elemental structures,^58^ these clusters could be well included in the category of intermetalloid compounds, as they represent a “growing class of metal-centred heteroatomic clusters”.^59^

Finally, the [Rh_21_Sb_2_(CO)_38_]^5–^ penta-anion shows a rich electrochemistry, as unravelled by cyclic-voltammetry and IR spectroelectrochemical studies. More specifically, the voltammetric profile of [Rh_21_Sb_2_(CO)_38_]^*n*−^ showed several reversible redox processes, which were also observed by tuning the working electrode potential with the *in situ* IR spectroelectrochemistry. These experimental results indicate that [Rh_21_Sb_2_(CO)_38_]^5–^ possesses multivalent properties, a feature shared with other carbonyl species of similar size. However, the low-intensity current in the CV study and the absence of isosbestic points in the IR SEC experiments prevented us from drawing incontrovertible conclusions on the number and charge of the oxidation states. Nevertheless, these studies further confirm the relevance of specific ad hoc conditions that favor multivalence, such as the presence of interstitial heteroatoms that strengthen the metal core, interstitial transition-metal atoms that may increase the number of molecular orbitals available for electrons, the high nuclearity, and the effectiveness of the ligand shielding.

## Supporting Information Available

IR spectra of **1**–**4**; ESI-MS spectra of **1**–**3**; ^31^P NMR spectrum of **4**; EDS analysis of **2**; CV, IR SEC spectra and deconvolution analyses of **3**; bond lengths from crystallographic analyses of **1**, **3**, and **4** (PDF)

## Accession Codes

CCDC 1960490–1960493 contain the supplementary crystallographic data for this paper. These data can be obtained free of charge via www.ccdc.cam.ac.uk/data_request/cif, or by emailing data_request@ccdc.cam.ac.uk, or by contacting The Cambridge Crystallographic Data Centre, 12 Union Road, Cambridge CB2 1EZ, UK; fax: +44 1223 336033.

## References

[ref1] Ciabatti I.; Femoni C.; Iapalucci M. C.; Ruggieri S.; Zacchini S. The role of gold in transition metal carbonyl clusters. Coord. Chem. Rev. 2018, 355, 27–38. 10.1016/j.ccr.2017.07.011.

[ref2] Femoni C.; Iapalucci M. C.; Ruggieri S.; Zacchini S. From Mononuclear Complexes to Molecular Nanoparticles: The Buildup of Atomically Precise Heterometallic Rhodium Carbonyl Nanoclusters. Acc. Chem. Res. 2018, 51, 2748–2755. 10.1021/acs.accounts.8b00354. 30346730

[ref3] Serna P.; Gates B. C. Zeolite-Supported Rhodium Complexes and Clusters Switching Catalytic Selectivity by Controlling Structures of Essentially Molecular Species. J. Am. Chem. Soc. 2011, 133, 4714–4717. 10.1021/ja111749s. 21391590

[ref4] Martinengo S.; Ciani G.; Sironi A. Synthesis and x-ray structural characterization of the [Rh_22_(μ_3_-CO)_7_(μ-CO)_18_(CO)_12_]^4-^ anion, containing a large closed-pack cluster with an ABAC sequence of compact layers. J. Am. Chem. Soc. 1980, 102, 7564–7565. 10.1021/ja00545a029.

[ref5] Dolzhnikov D. S.; Iapalucci M. C.; Longoni G.; Tiozzo C.; Zacchini S.; Femoni C. New High-Nuclearity Carbonyl and Carbonyl-Substituted Rhodium Clusters and Their Relationships with Polyicosahedral Carbonyl-Substituted Palladium- and Gold-Thiolates. Inorg. Chem. 2012, 51, 11214–11216. 10.1021/ic3011508. 23043503

[ref6] Wade K. Metal-metal and metal-carbon bond energy terms for the rhodium carbonyl clusters Rh_4_(CO)_12_ and Rh_6_(CO)_16_. Inorg. Nucl. Chem. Lett. 1978, 14, 71–74. 10.1016/0020-1650(78)80032-4.

[ref7] Hughes A. K.; Wade K. Metal-metal and metal-ligand bond strengths in metal carbonyl clusters. Coord. Chem. Rev. 2000, 197, 191–229. 10.1016/S0010-8545(99)00208-8.

[ref8] Collini D.; Fedi S.; Femoni C.; Kaswalder F.; Iapalucci M. C.; Longoni G.; Zanello P. New Bimetallic Ni-Rh Carbonyl Clusters: Synthesis and X-ray Structure of the [Ni_7_Rh_3_(CO)_18_]^3-^, [Ni_3_Rh_3_(CO)_13_]^3-^ and [NiRh_8_(CO)_19_]^2-^ Cluster Anions. J. Cluster Sci. 2005, 16, 455–476. 10.1007/s10876-005-0014-0.

[ref9] Collini D.; Femoni C.; Iapalucci M. C.; Longoni G.; Svensson P. H.; Zanello P. Tuning Electronic Behavior of Carbonyl Metal Clusters by Substitution of Interstitial and Capping Atoms. Angew. Chem., Int. Ed. 2002, 41, 3685–3688. 10.1002/1521-3773(20021004)41:19<3685::AID-ANIE3685>3.0.CO;2-7. 12370931

[ref10] Femoni C.; Bussoli G.; Ciabatti I.; Ermini M.; Hayatifar M.; Iapalucci M. C.; Ruggieri S.; Zacchini S. Interstitial Bismuth Atoms in Icosahedral Rhodium Cages: Syntheses, Characterizations, and Molecular Structures of the [Bi@Rh_12_(CO)_27_]^3-^, [(Bi@Rh_12_(CO)_26_)_2_Bi]^5-^, [Bi@Rh_14_(CO)_27_Bi_2_]^3-^, and [Bi@Rh_17_(CO)_33_Bi_2_]^4-^ Carbonyl Clusters. Inorg. Chem. 2017, 56, 6343–6351. 10.1021/acs.inorgchem.7b00409. 28520423

[ref11] Longoni G.; Femoni C.; Iapalucci M. C.; Zanello P.; Braunstein P.; Oro L. A.; Raithby P. R. Electron-Sink Features of Homoleptic Transition-Metal Carbonyl Clusters. Metal Clusters in Chemistry 1999, 2, 1137–1158. 10.1002/9783527618316.ch3i.

[ref12] Fumagalli A.; Martinengo S.; Bernasconi G.; Noziglia L.; Albano V. G.; Monari M.; Castellari C. Skeletal Growth by Condensation of Small Metal Fragments on a Carbido Carbonyl Cluster. Synthesis of the Anions [Rh_15_C_2_(CO)_24_X_2_]^3-^ (X = Cl, Br, I) and Molecular Structure of the Bromo and Iodo Derivatives. Organometallics 2000, 19, 5149–5154. 10.1021/om000577p.

[ref13] Fumagalli A.; Martinengo S.; Bernasconi G.; Ciani G.; Proserpio D. M.; Sironi A. [Rh_28_N_4_(CO)_41_H_x_]^4-^, a Massive Carbonyl Cluster with Four Interstitial Nitrogen Atoms. J. Am. Chem. Soc. 1997, 119, 1450–1451. 10.1021/ja961403v.

[ref14] Vidal J. L.; Walker W. E.; Schoening R. C. [Rh_10_P(CO)_22_]^3-^. A transition-metal carbonyl cluster with a metal polyhedron based on the bicapped square antiprism as illustrated by the structural study of the benzyltriethylammonium salt. Inorg. Chem. 1981, 20, 238–242. 10.1021/ic50215a047.

[ref15] aCiani G.; Garlaschelli L.; Sironi A.; Martinengo S. Synthesis and X-ray characterization of the novel [Rh_10_S(CO)_10_(μ-CO)_12_]^2-^ anion; a bicapped square-antiprismatic cluster containing an interstitial sulphur atom. J. Chem. Soc., Chem. Commun. 1981, 0, 563b–565. 10.1039/C3981000563B.

[ref16] Boccalini A.; Dyson P. J.; Femoni C.; Iapalucci M. C.; Ruggieri S.; Zacchini S. Insertion of germanium atoms in high-nuclearity rhodium carbonyl compounds: synthesis, characterization and preliminary biological activity of the heterometallic [Rh_13_Ge(CO)_25_]^3-^, [Rh_14_Ge_2_(CO)_30_]^2-^ and [Rh_12_Ge(CO)_27_]^4-^ cluster anions. Dalton Trans. 2018, 47, 15737–15744. 10.1039/C8DT02466A. 30106077

[ref17] Femoni C.; Iapalucci M. C.; Longoni G.; Tiozzo C.; Zacchini S.; Heaton B. T.; Iggo J. A. Sn-centred icosahedral Rh carbonyl clusters: synthesis and structural characterization and ^13^C-{^103^Rh} HMQC NMR studies. Dalton Trans. 2007, 35, 3914–3923. 10.1039/b708469b. 17893789

[ref18] Femoni C.; Iapalucci M. C.; Longoni G.; Tiozzo C.; Zacchini S.; Heaton B. T.; Iggo J. A.; Zanello P.; Fedi S.; Garland M. V.; Li C. The loss of CO from [Rh_12_(μ_12_-Sn)(CO)_27_]^4-^: Synthesis, spectroscopic and structural characterization of the electron-deficient, icosahedral [Rh_12_(μ_12_-Sn)(CO)_25_]^4-^ and [Rh_12_(μ_12_-Sn)(CO)_26_]^4-^ tetra-anions. Dalton Trans. 2009, 2217–2223. 10.1039/b818944g. 19274301

[ref19] Vidal J. L.; Troup J. M. [Rh_12_Sb(CO)_27_]^3-^. An example of encapsulation of antimony by a transition metal carbonyl cluster. J. Organomet. Chem. 1981, 213, 351–363. 10.1016/S0022-328X(00)93970-6.

[ref20] Femoni C.; Ciabatti I.; Iapalucci M. C.; Ruggieri S.; Zacchini S. Alternative synthetic route for the heterometallic CO-releasing [Sb@Rh_12_(CO)_27_]^3-^ icosahedral carbonyl cluster and synthesis of its new unsaturated [Sb@Rh_12_(CO)_24_]^4-^ and dimeric [{Sb@Rh_12_Sb(CO)_25_}_2_Rh(CO)_2_PPh_3_]^7-^ derivatives. Prog. Nat. Sci. 2016, 26, 461–466. 10.1016/j.pnsc.2016.08.004.

[ref21] Kim S.-G.; Dhandole L. K.; Seo Y.-S.; Chung H.-S.; Chae W.-S.; Cho M.; Jang J. S. Active composite photocatalyst synthesized from inactive Rh & Sb doped TiO_2_ nanorods: Enhanced degradation of organic pollutants & antibacterial activity under visible light irradiation. Appl. Catal., A 2018, 564, 43–55. 10.1016/j.apcata.2018.07.016.

[ref22] Zacchini S. Using Metal Carbonyl Clusters To Develop a Molecular Approach towards Metal Nanoparticles. Eur. J. Inorg. Chem. 2011, 2011, 4125–4145. 10.1002/ejic.201100462.

[ref23] Albano V. G.; Demartin F.; Femoni C.; Iapalucci M. C.; Longoni G.; Monari M.; Zanello P. Synthesis and characterization of new paramagnetic nickel carbonyl clusters containing antimony atoms: X-ray structure of [NEt_3_CH_2_Ph]_2_[Ni_15_(μ_12_-Sb)(CO)_24_] and [NEt_4_]_3_[Ni_10_Sb_2_(μ_12_-Ni)(CO)_18_]. J. Organomet. Chem. 2000, 593, 325–334. 10.1016/S0022-328X(99)00523-9.

[ref24] Mlynek P. D.; Dahl L. F. New Nickel-Antimony Carbonyl Clusters: Stereochemical Analyses of the [Ni_10_(SbR)_2_(CO)_18_]^2-^ Dianions (R = Me, Et, *^i^*Pr, *^t^*Bu, *p*-FC_6_H_4_) Containing Empty 1,12-Ni_10_Sb_2_ Icosahedral Cages and of the Unprecedented Stibinido-Bridged 34-Electron Ni_2_(CO)_4_(μ_2_-Sb*^t^*Bu_2_)_2_ Dimer^1^. Organometallics 1997, 16, 1641–1654. 10.1021/om960966c.

[ref25] Femoni C.; Iapalucci M. C.; Longoni G.; Svensson P. H. A high-nuclearity Ni-Sb carbonyl cluster displaying unprecedented metal stereochemistries: synthesis and X-ray structure of [NEt_4_]_6_[Ni_31_Sb_4_(CO)_40_]·2 Me_2_CO. Chem. Commun. 2000, 8, 655–656. 10.1039/b000783h.

[ref26] Li Y.-Z.; Ganguly R.; Leong W. K. Ligand substitution in the osmium-antimony rings Os_3_(μ-SbPh_2_)_2_(CO)_10_ and Os_3_(μ-SbPh_2_)_3_(Cl)(CO)_9_. J. Organomet. Chem. 2016, 820, 46–54. 10.1016/j.jorganchem.2016.07.028.

[ref27] Li Y.-Z.; Leong W. K. Raft-like osmium- and ruthenium-antimony carbonyl clusters. J. Organomet. Chem. 2016, 812, 217–225. 10.1016/j.jorganchem.2015.06.007.

[ref28] Chen G.; Leong W. K. Two Group 8 Carbonyl Clusters Containing a Naked μ_5_-Sb Atom. J. Cluster Sci. 2006, 17, 111–118. 10.1007/s10876-005-0035-8.

[ref29] Hieber W. O.; Schubert E. H. Absorptionsmessungen an Carbonylferrat-Lösungen im Sichtbaren und UV-Gebiet. Z. Anorg. Allg. Chem. 1965, 338, 32–36. 10.1002/zaac.19653380106.

[ref30] Chini P. Large Metal Carbonyl Clusters (LMCC). J. Organomet. Chem. 1980, 200, 37–61. 10.1016/S0022-328X(00)88636-2.

[ref31] Martinengo S.; Chini P. Synthesis and characterization of the [Rh_6_(CO)_15_]^2-^ and [Rh_7_(CO)_16_]^3-^ anions. Gazz. Chim. It. 1972, 102, 344–354.

[ref32] Ceriotti A.; Longoni G.; Manassero M.; Masciocchi N.; Piro G.; Resconi L.; Sansoni M. Synthesis and X-ray structure of [Ni_16_(CO)_23_C_4_]^4-^: a tetracarbide anionic cluster containing two interstitial C_2_ fragments. J. Chem. Soc., Chem. Commun. 1985, 1402–1403. 10.1039/C39850001402.

[ref33] Tunik S.P.; Vlasov A.V.; Kogdov K.V.; Starova G.L.; Nikol'skii A.B.; Manole O.S.; Struchkov Yu.T. Synthesis and structural characterization of the isomers of Rh_6_(CO)_14_L_2_ clusters (L = NCME, Py, P(OPh)_3_), X-ray crystal structure of *trans*-Rh_6_(CO)_14_{P(OPh_3_)}_2_. J. Organomet. Chem. 1994, 479, 59–72. 10.1016/0022-328X(94)84092-X.

[ref34] Mingos D. M. P. Polyhedral skeletal electron pair approach. Acc. Chem. Res. 1984, 17, 311–319. 10.1021/ar00105a003.

[ref35] Shriver D. F., Herbert D. K., Adams R. D., Eds. The Chemistry of Metal Cluster Complexes; VCH: New York, 1990.

[ref36] Collini D.; Femoni C.; Iapalucci M. C.; Longoni G.; Zanello P. Modulation of electronic behaviour of metal carbonyl clusters. Perspectives in Organometallic Chemistry 2007, 287, 183–195. 10.1039/9781847551641-00183.

[ref37] Femoni C.; Iapalucci M. C.; Kaswalder F.; Longoni G.; Zacchini S. The possible role of metal carbonyl clusters in nanoscience and nanotechnologies. Coord. Chem. Rev. 2006, 250, 1580–1604. 10.1016/j.ccr.2006.03.011.

[ref38] Nair L. V.; Hossain S.; Takagi S.; Imai Y.; Hu G.; Wakayama S.; Kumar B.; Kurashige W.; Jiang D.; Negishi Y. Hetero-biicosahedral [Au_24_Pd(PPh_3_)_10_(SC_2_H_4_Ph)_5_Cl_2_]^+^ nanocluster: selective synthesis and optical and electrochemical properties. Nanoscale 2018, 10, 18969–18979. 10.1039/C8NR04078H. 30132774

[ref39] Capacci C.; Ciabatti I.; Femoni C.; Iapalucci M. C.; Funaioli T.; Zacchini S.; Zanotti V. Molecular Nickel Phosphide Carbonyl Nanoclusters: Synthesis, Structure, and Electrochemistry of [Ni_11_P(CO)_18_]^3-^ and [H_6-n_Ni_31_P_4_(CO)_39_]^n-^ (n = 4 and 5). Inorg. Chem. 2018, 57, 1136–1147. 10.1021/acs.inorgchem.7b02598. 29303559

[ref40] Calderoni F.; Demartin F.; Fabrizi de Biani F.; Femoni C.; Iapalucci M. C.; Longoni G.; Zanello P. Electron-Sink Behaviour of the Carbonylnickel Clusters [Ni_32_C_6_(CO)_36_]^6-^ and [Ni_38_C_6_(CO)_42_]^6-^: Synthesis and Characterization of the Anions [Ni_32_C_6_(CO)_36_]^n-^ (n = 5–10) and [Ni_38_C_6_(CO)_42_]^n-^ (n = 5–9) and Crystal Structure of [PPh_3_Me]_6_[Ni_32_C_6_(CO)_36_]·4MeCN. Eur. J. Inorg. Chem. 1999, 1999, 663–671. 10.1002/(SICI)1099-0682(199904)1999:4<663::AID-EJIC663>3.0.CO;2-1.

[ref41] Krejčik M.; Daněk M.; Hartl F. Simple construction of an infrared optically transparent thin-layer electrochemical cell: Applications to the redox reactions of ferrocene, Mn_2_(CO)_10_ and Mn(CO)_3_(3,5-di-t-butyl-catecholate)^−^. J. Electroanal. Chem. Interfacial Electrochem. 1991, 317, 179–187. 10.1016/0022-0728(91)85012-E.

[ref42] Roth J. D.; Lewis G. J.; Safford L. K.; Jiang X.; Dahl L. F.; Weaver M. J. Exploration of the Ionizable Metal Cluster-Electrode Surface Analogy: Infrared Spectroelectrochemistry of [Pt_24_(CO)_30_]^n^, [Pt_26_(CO)_32_]^n^, and [Pt_38_(CO)_44_]^n^ (*n* = 0 to −10) and Comparisons with Potential-Dependent Spectra of CO Adlayers on Platinum Surfaces. J. Am. Chem. Soc. 1992, 114, 6159–6169. 10.1021/ja00041a038.

[ref43] Collini D.; Fabrizi de Biani F.; Dolzhnikov D. S.; Femoni C.; Iapalucci M. C.; Longoni G.; Tiozzo C.; Zacchini S.; Zanello P. Synthesis, Structure, and Spectroscopic Characterization of [H_8-n_Rh_22_(CO)_35_]^n-^ (n = 4, 5) and [H_2_Rh_13_(CO)_24_{Cu(MeCN)}_2_]^−^ Clusters: Assessment of CV and DPV As Techniques to Circumstantiate the Presence of Elusive Hydride Atoms. Inorg. Chem. 2011, 50, 2790–2798. 10.1021/ic101872z. 21314145

[ref44] Cattabriga E.; Ciabatti I.; Femoni C.; Funaioli T.; Iapalucci M. C.; Zacchini S. Syntheses, Structures, and Electrochemistry of the Defective ccp [Pt_33_(CO)_38_]^2-^ and the bcc [Pt_40_(CO)_40_]^6-^ Molecular Nanoclusters. Inorg. Chem. 2016, 55, 6068–6079. 10.1021/acs.inorgchem.6b00607. 27281686

[ref45] Fedi S.; Zanello P.; Laschi F.; Ceriotti A.; El Afefey S. J. Solid State Electrochem. 2009, 13, 1497–1504. 10.1007/s10008-009-0880-8.

[ref46] Fabrizi de Biani F.; Femoni C.; Iapalucci M. C.; Longoni G.; Zanello P.; Ceriotti A. Redox Behavior of [H_6-*n*_Ni_38_Pt_6_(CO)_48_]*^n-^* (*n* = 4–6) Anions: A Series of Metal Carbonyl Clusters Displaying Electron-Sink Features. Inorg. Chem. 1999, 38, 3721–3724. 10.1021/ic9813516. 11671133

[ref47] Sheldrick G. M. SADABS, Program for empirical absorption correction; University of Göttingen: Germany, 1996.

[ref48] Sheldrick G. M. SHELX 2014/7, Program for crystal structure determination; University of Göttingen: Germany, 2014.

[ref49] Keller E. SCHAKAL99; University of Freiburg: Germany, 1999.

[ref50] Spek A. L. PLATON SQUEEZE: a tool for the calculation of the disordered solvent contribution to the calculated structure factors. Acta Crystallogr., Sect. C: Struct. Chem. 2015, C71, 9–18. 10.1107/S2053229614024929. 25567569

[ref51] Bernardi A.; Femoni C.; Iapalucci M. C.; Longoni G.; Zacchini S. The problems of detecting hydrides in metal carbonyl clusters by ^1^H NMR: the case study of [H_4-*n*_Ni_22_(C_2_)_4_(CO)_28_(CdBr)_2_]*^n-^* (*n* = 2–4). Dalton Trans. 2009, 21, 4245–4251. 10.1039/b900950g. 19452075

[ref52] Zeng C.; Liu C.; Pei Y.; Jin R. Thiol Ligand-Induced Transformation of Au_38_(SC_2_H_4_Ph)_24_ to Au_36_(SPh-t-Bu)_24_. ACS Nano 2013, 7, 6138–6145. 10.1021/nn401971g. 23758648

[ref53] Yan N.; Xia N.; Liao L.; Zhu M.; Jin F.; Jin R.; Wu Z. Unraveling the long-pursued Au_144_ structure by x-ray crystallography. Sci. Adv. 2018, 4, eaat7259 10.1126/sciadv.aat7259. 30333988 PMC6184749

[ref54] Desireddy A.; Conn B. E.; Guo J.; Yoon B.; Barnett R. N.; Monahan B. M.; Kirschbaum K.; Griffith W. P.; Whetten R. L.; Landman U.; Bigioni T. P. Ultrastable silver nanoparticles. Nature 2013, 501, 399–402. 10.1038/nature12523. 24005327

[ref55] Erickson J. D.; Mednikov E. G.; Ivanov S. A.; Dahl L. F. Isolation and Structural Characterization of a Mackay 55-Metal-Atom Two-Shell Icosahedron of Pseudo-I_h_ Symmetry, Pd_55_L_12_(μ_3_-CO)_20_ (L = PR_3_, R = Isopropyl): Comparative Analysis with Interior Two-Shell Icosahedral Geometries in Capped Three-Shell Pd_145_, Pt-Centered Four-Shell Pd-Pt M_165_, and Four-Shell Au_133_ Nanoclusters. J. Am. Chem. Soc. 2016, 138, 1502–1505. 10.1021/jacs.5b13076. 26790717

[ref56] Mednikov E. G.; Jewell M. C.; Dahl L. F. Nanosized (μ_12_-Pt)Pd_164–*x*_Pt_x_(CO)_72_(PPh_3_)_20_ (x ≍ 7) Containing Pt-Centered Four-Shell 165-Atom Pd-Pt Core with Unprecedented Intershell Bridging Carbonyl Ligands: Comparative Analysis of Icosahedral Shell-Growth Patterns with Geometrically Related Pd_145_(CO)_x_(PEt_3_)_30_ (x ≍ 60) Containing Capped Three-Shell Pd_145_ Core. J. Am. Chem. Soc. 2007, 129, 11619–11630. 10.1021/ja073945q. 17722929

[ref57] Tran N. T.; Powell D. R.; Dahl L. F. Nanosized Pd_145_(CO)_x_(PEt_3_)_30_ Containing a Capped Three-Shell 145-Atom Metal-Core Geometry of Pseudo Icosahedral Symmetry. Angew. Chem., Int. Ed. 2000, 39, 4121–4125. 10.1002/1521-3773(20001117)39:22<4121::AID-ANIE4121>3.0.CO;2-A. 11093227

[ref58] Schnepf A.; Schnockel H. Metalloid Aluminum and Gallium Clusters: Element Modifications on the Molecular Scale?. Angew. Chem., Int. Ed. 2002, 41, 3532–3554. 10.1002/1521-3773(20021004)41:19<3532::AID-ANIE3532>3.0.CO;2-4. 12370894

[ref59] Fässler T. F.; Hoffmann S. D. Endohedral Zintl Ions: Intermetalloid Clusters. Angew. Chem., Int. Ed. 2004, 43, 6242–6247. 10.1002/anie.200460427. 15505810

