# Supplementary material for: Rh–Sb Nanoclusters: Synthesis, Structure, and Electrochemical Studies of the Atomically Precise [Rh20Sb3(CO)36]3– and [Rh21Sb2(CO)38]5– Carbonyl Compounds
Source: Inorg Chem. 2020 Mar 24;59(7):4300–10. doi: 10.1021/acs.inorgchem.9b03135 (PMC7997401; doi:10.1021/acs.inorgchem.9b03135)

Rh-Sb Nanoclusters: Synthesis, Structure, and Electrochemical Studies of the Atomically Precise $[Rh_{20}Sb_3(CO)_{36}]^{3-}$ and $[Rh_{21}Sb_2(CO)_{38}]^{5-}$ Carbonyl Compounds

Cristina Femoni,*[†] Tiziana Funaioli,[‡] Maria Carmela Iapalucci,[†] Silvia Ruggieri,*[†] Stefano Zacchini[†]
[†] Dipartimento di Chimica Industriale "Toso Montanari", Università di Bologna, Viale del Risorgimento, 4 – Bologna, Italy
[‡] Dipartimento di Chimica e Chimica Industriale, Università di Pisa, Via Moruzzi 13, 56124 Pisa, Italy
* cristina.femoni@unibo.it; silvia.ruggieri3@unibo.it

SUPPORTING INFORMATION

LIST OF CONTENT

Cluster 1.
IR spectrum (Figure S1)
ESI–MS spectra and table (Figure S2 and Table S1)

Cluster 2.
IR spectrum (Figure S3)
SEM image and EDS spectrum (Figure S4) and table (Table S2)
ESI–MS spectrum and table (Figure S5 and Table S3)

Cluster 3.
IR spectrum (Figure S6)
ESI–MS spectrum and table (Figure S7 and Table S4)
CV experiments (Figure S8 and S9)
IR SEC spectra and deconvolution analyses (Figures S10 – S12)

Cluster 4.
IR spectrum (Figure S13)
^{31}P NMR spectrum (Figure S14)

Clusters 1, 3 and 4.
Bond lengths from crystallographic analyses with labelled metal skeletons.

IR and ESI mass spectra for $[Rh_{20}Sb_3(CO)_{36}]^{3-}$ (1).

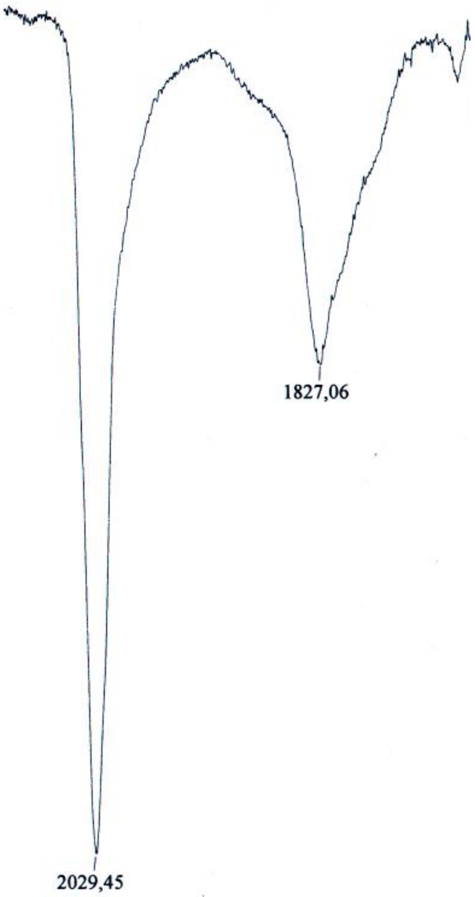

Figure S1. IR spectrum of $[Rh_{20}Sb_3(CO)_{36}][NEt_4]_3 \cdot 2(CH_3)_2CO$ registered in CH_3CN solution.

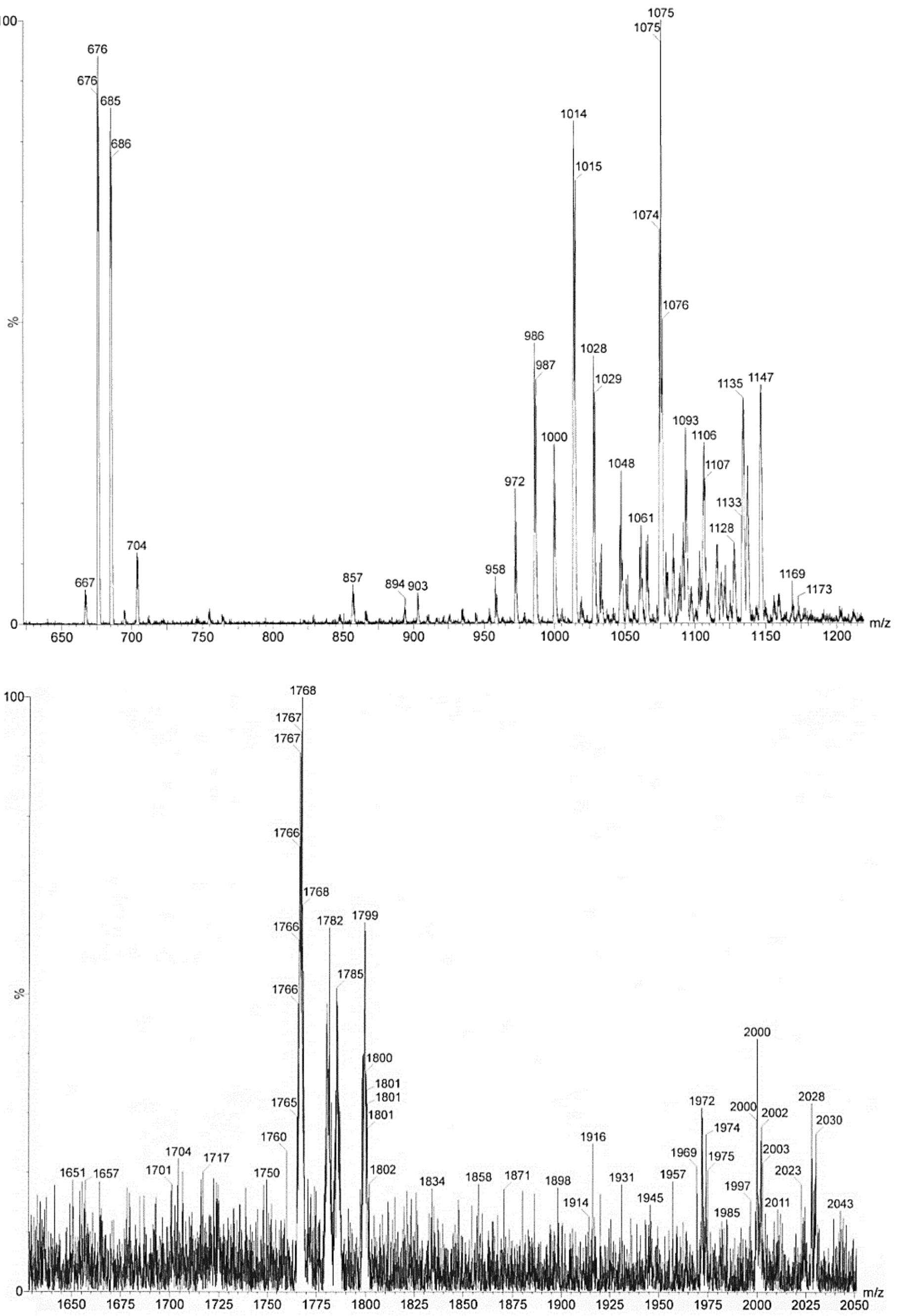

Figure S2. ESI–MS of $[Rh_{20}Sb_3(CO)_{36}][NEt_4]_3 \cdot 2(CH_3)_2CO$ registered in CH_3CN solution.

**Most relevant signals (m/z)**	**Corresponding Ions**
2028-2000-1972-1945-1916	$\{[Rh_{10}Sb_3(CO)_{18\text{-}17\text{-}16\text{-}15\text{-}14}][NEt_4]\}^{-}$
1782-1768	$\{[Rh_{20}Sb_3(CO)_{36\text{-}35}][NEt_4]\}^{2-}$
1135-1106	$\{[Rh_{20}Sb_3(CO)_{35\text{-}32}]\}^{3-}$
1075-1061-1048	$\{[Rh_{10}Sb_2(CO)_{22\text{-}21\text{-}20}][NEt_4]_2\}^{2-}$
1028-1014-1000-986-972-958	$\{[Rh_{10}Sb_3(CO)_{19\text{-}18\text{-}17\text{-}16\text{-}15\text{-}14}][NEt_4]\}^{2-}$
704-685-676	$\{[Rh_{10}Sb_3(CO)_{21\text{-}19\text{-}18}][NEt_4]\}^{3-}$

Table S1. ESI–MS peak assignments for $[Rh_{20}Sb_3(CO)_{36}][NEt_4]_3 \cdot 2(CH_3)_2CO$.

IR, EDS and ESI–MS spectra for $[Rh_{28-x}Sb_x(CO)_{44}]^{6-}$ (2).

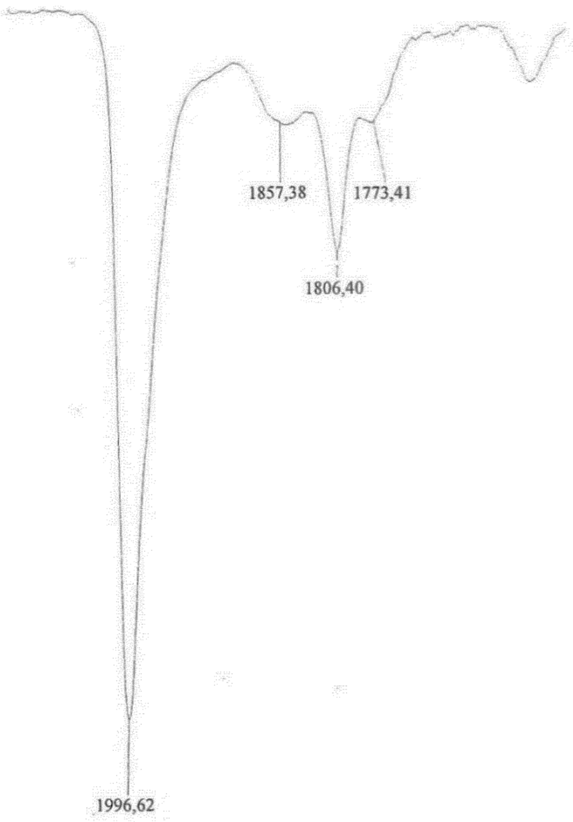

Figure S3. IR spectrum of $[Rh_{28-x}Sb_x(CO)_{44}][NEt_4]_6$ registered in CH_3CN solution.

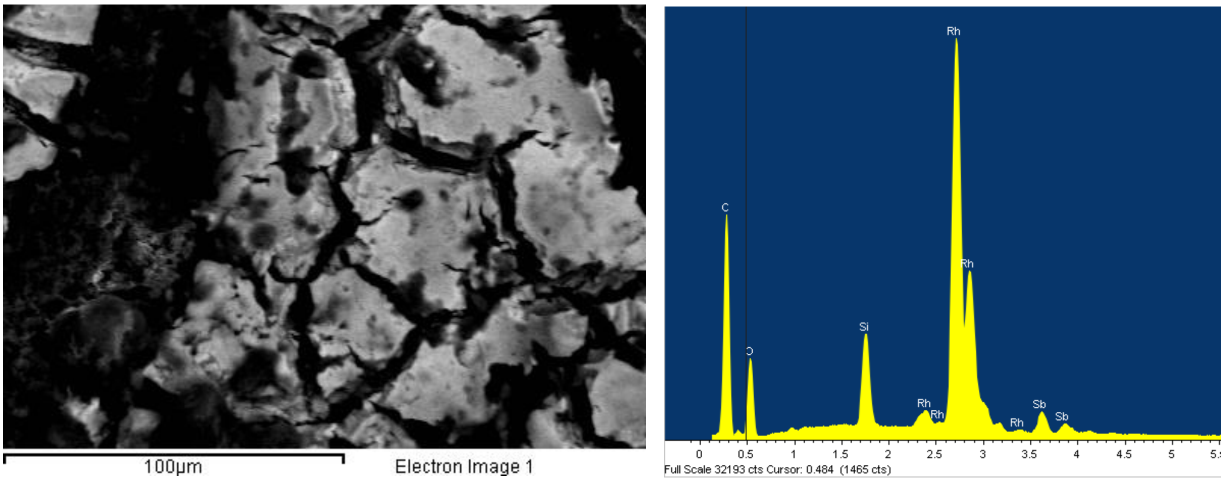

Figure S4. SEM image and EDS spectrum of $[Rh_{28-x}Sb_x(CO)_{44}][NEt_4]_6$.

Spectrum	1	2	3	4	5	6	7	Mean	Std Dev.
Rh (%)	0,913504	0,922644	0,922164	0,9177	0,917673	0,917088	0,916747	0,918217	0,003199
Sb (%)	0,086496	0,077356	0,077836	0,0823	0,082327	0,082912	0,083253	0,081783	0,003199

Table S2. EDS analyses on different areas of a selected crystal of cluster **2**.

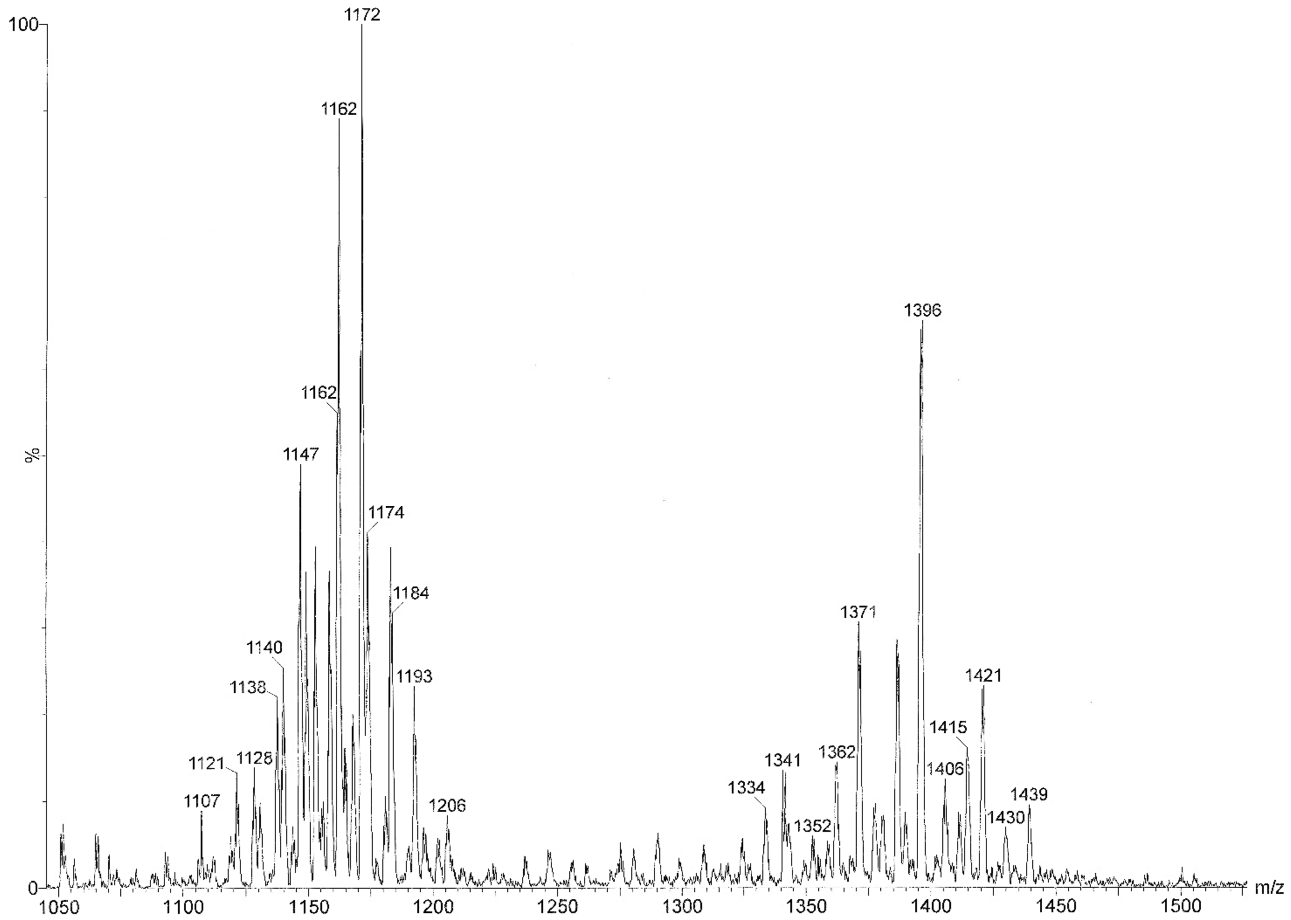

Figure S5. ESI-MS of $[Rh_{28-x}Sb_x(CO)_{44}][NMe_4]_6$ registered in CH_3CN solution.

**Most relevant signals (m/z)**	**Corresponding Ions**
1439-1430-1421	$\{[Rh_{25}Sb_3(CO)_{44\text{-}43\text{-}42}][NMe_4]_2\}^{3-}$
1415-1406-1396	$\{[Rh_{25}Sb_3(CO)_{44\text{-}43\text{-}42}][NMe_4]\}^{3-}$
1371-1362	$\{[Rh_{25}Sb_3(CO)_{42\text{-}41}]\}^{3-}$
1172-1162-1153	$\{[Rh_{21}Sb_2(CO)_{37\text{-}36\text{-}35}][NMe_4]\}^{3-}$
1147-1138	$\{[Rh_{21}Sb_2(CO)_{37\text{-}36}]\}^{3-}$

Table S3. ESI–MS peak assignments for $[Rh_{28-x}Sb_x(CO)_{44}][NMe_4]_6$.
The last two lines describe the signals due to the presence of cluster **3** in the analysed sample.

IR and ESI mass spectra for $[Rh_{21}Sb_2(CO)_{38}]^{5-}$ (3).

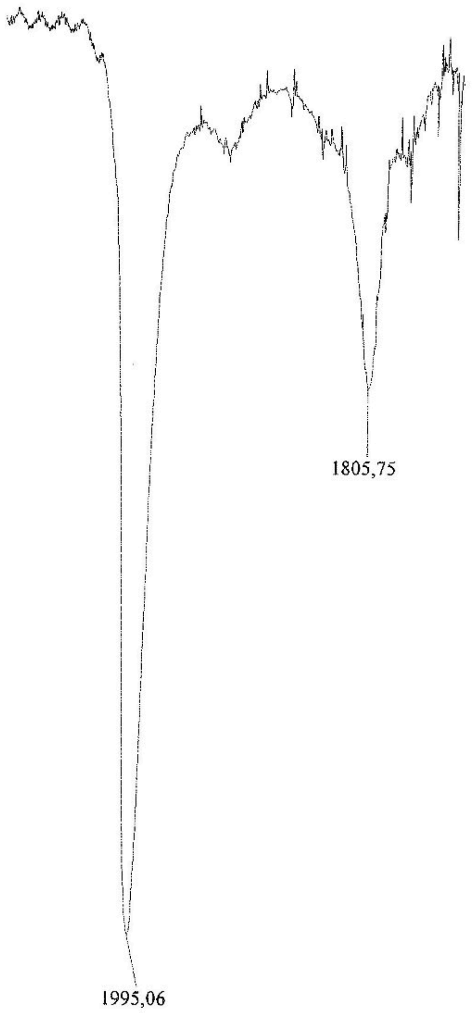

Figure S6. IR spectrum of $[Rh_{21}Sb_2(CO)_{38}][NEt_4]_5 \cdot 4CH_3CN$.registered in CH_3CN solution.

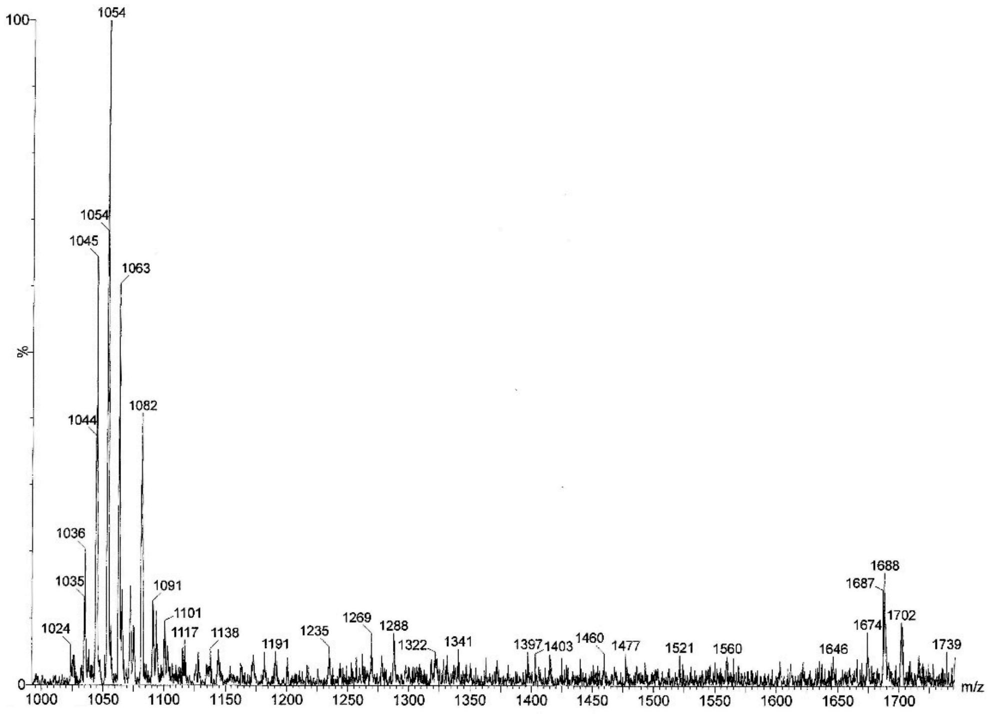

Figure S7. ESI–MS of $[Rh_{21}Sb_2(CO)_{38}][NEt_4]_5 \cdot 4CH_3CN$ registered in CH_3CN solution.

**Most relevant signals (m/z)**	**Corresponding Ions**
1716-1702-1688-1674-1660-1646	$\{[Rh_{21}Sb_2(CO)_{32\text{-}31\text{-}30\text{-}29\text{-}28\text{-}27}][NEt_4]\}^{2-}$
1101-1091-1082-1073-1063-1054-1045-1036	$\{[Rh_{21}Sb_2(CO)_{32\text{-}31\text{-}30\text{-}29\text{-}28\text{-}27\text{-}26\text{-}25}]\}^{3-}$

Table S4. ESI–MS peak assignments for $[Rh_{21}Sb_2(CO)_{38}][NEt_4]_5 \cdot 4CH_3CN$.

Electrochemical and spectroelectrochemical studies of $[Rh_{21}Sb_2(CO)_{38}]^{5-}$ (3).

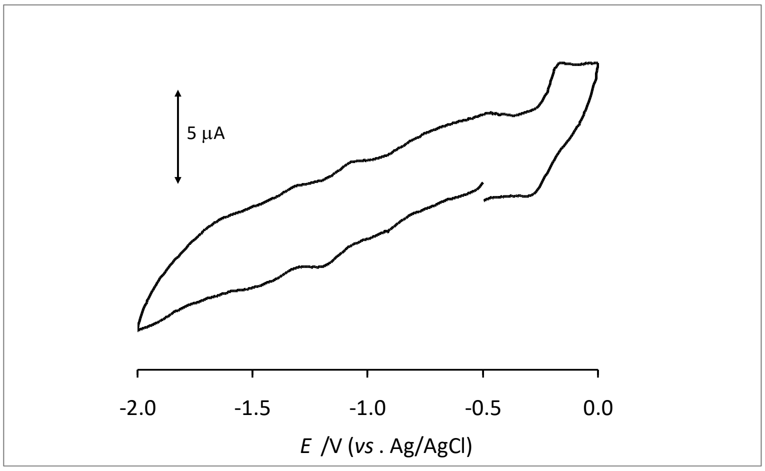

Figure S8. Cyclic voltammetric profile recorded at Pt electrode in CH_3CN solution of **3**. $[N^nBu_4][PF_6]$ (0.1 mol dm^{-3}) supporting electrolyte. Scan rate: 0.2 V s^{-1}.

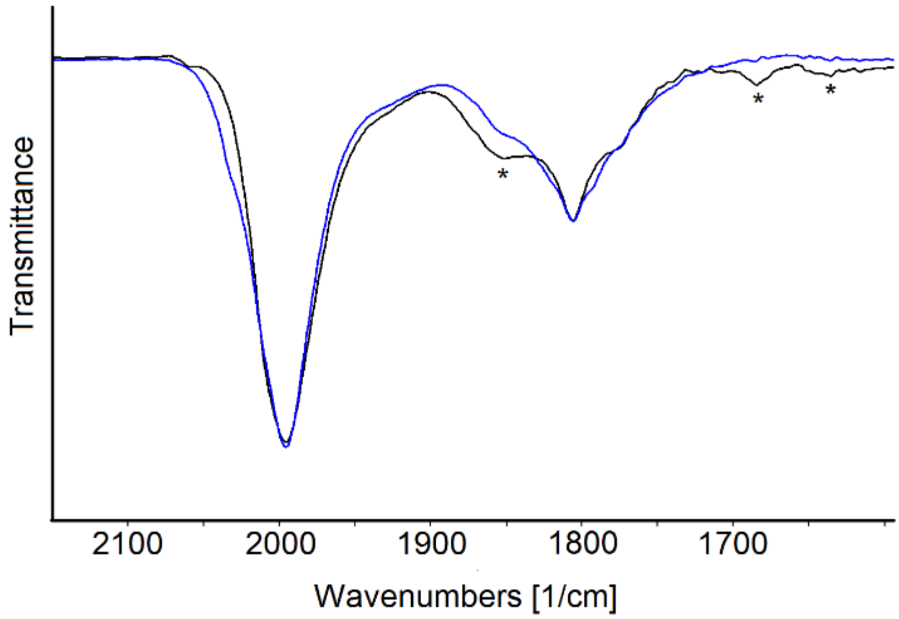

Figure S9. Comparison between the IR spectra of a CH_3CN solution of $[Rh_{21}Sb_2(CO)_{38}]^{5-}$ recorded in an OTTLE cell before (blue line) and after (black line) a cyclic voltammetry between –0.6 to –2.1 V *vs.* Ag pseudoreference electrode (scan rate 1mV s^{-1}). $[N^nBu_4][PF_6]$ (0.1 mol dm^{-3}) as the supporting electrolyte. The absorptions of the solvent and the supporting electrolyte have been subtracted.

IR-SEC spectra and deconvolution analyses.

In the oxidation sequence, between the two absorptions at 2026 and 1996 cm^{-1} of Figure S10 (left), the band at 2012 cm^{-1} (light green line) did not correspond to an absorbance maximum. In order to determine its single contribution, we performed a spectral deconvolution by comparing the curve fitting analysis of three consecutive oxidation spectra. The results showed that those curves are matched by the same three individual component bands at 2026, 1996 and 2012 cm^{-1}, but in different ratios. In particular, the 2012 cm^{-1} band is always the predominant one, whereas the other two vary their relative intensity according to the direction of the potential scan (Figure S11). In light of this, we assigned the peak at 2012 cm^{-1} to a different and intermediate oxidation state.

We performed the same spectral deconvolution onto the reduction sequence spectra (Figure S12), and determined that the orange curve of Figure S10 (right) could be fitted by four components, of which the one at 1944 cm^{-1} to be attributed to a new and hidden redox state of the cluster.

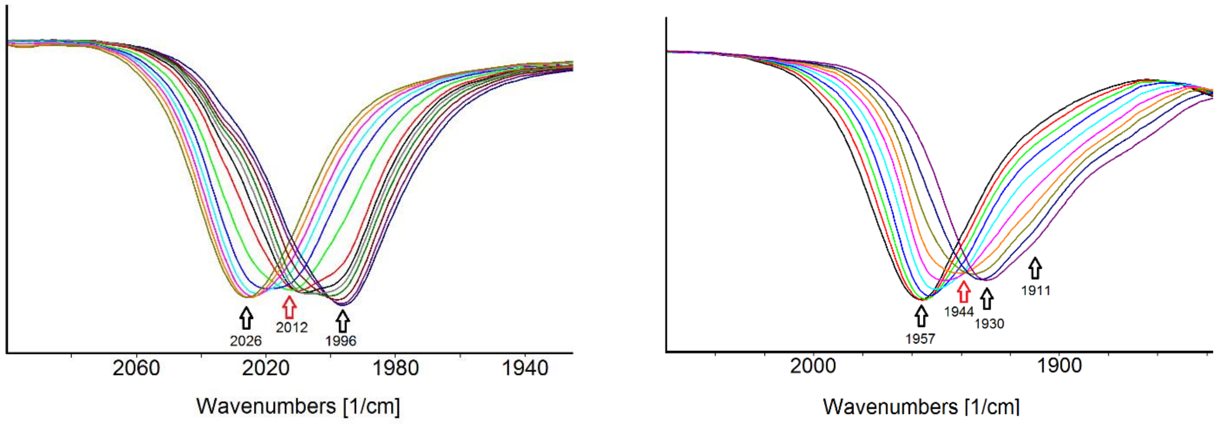

Figure S10. IR spectral changes of a CH_3CN solution of **3** recorded in an OTTLE cell during the progressive: (left) increase of the potential from –0.6 to +0.6 V, (right) decrease of the potential from –1.32 to –1.90 V *vs.* Ag pseudoreference electrode (scan rate 1mV s^{-1}).

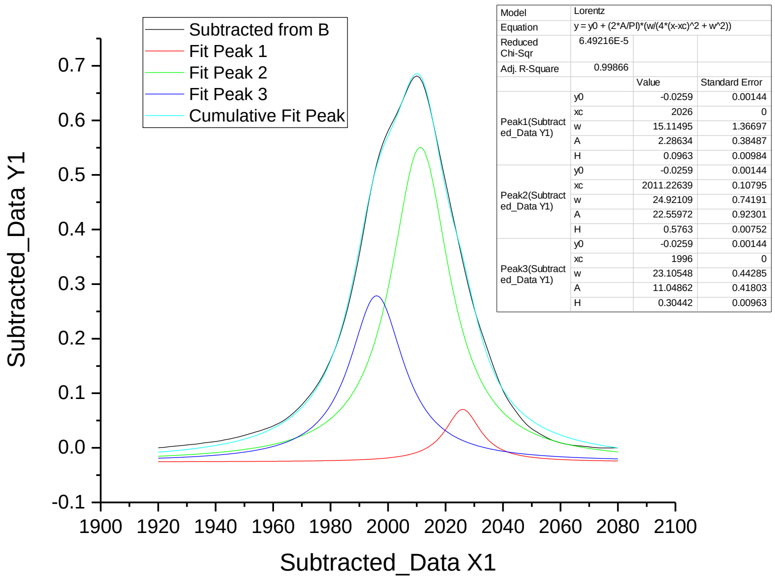

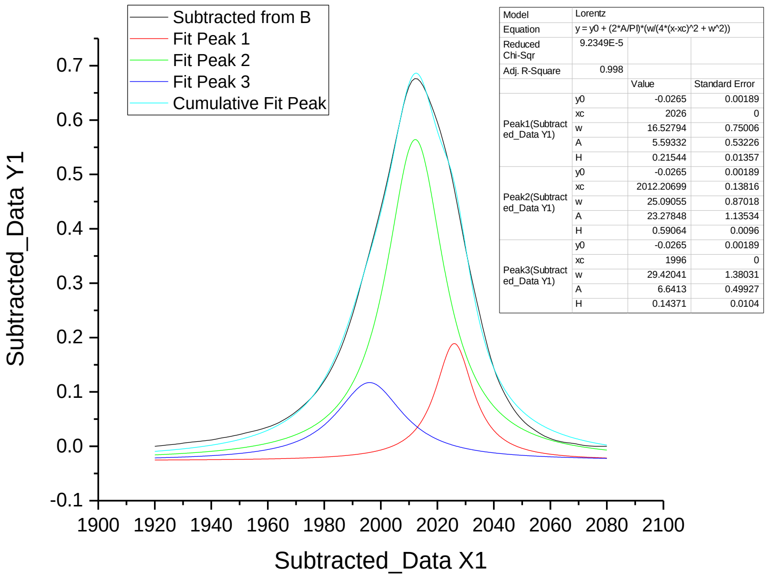

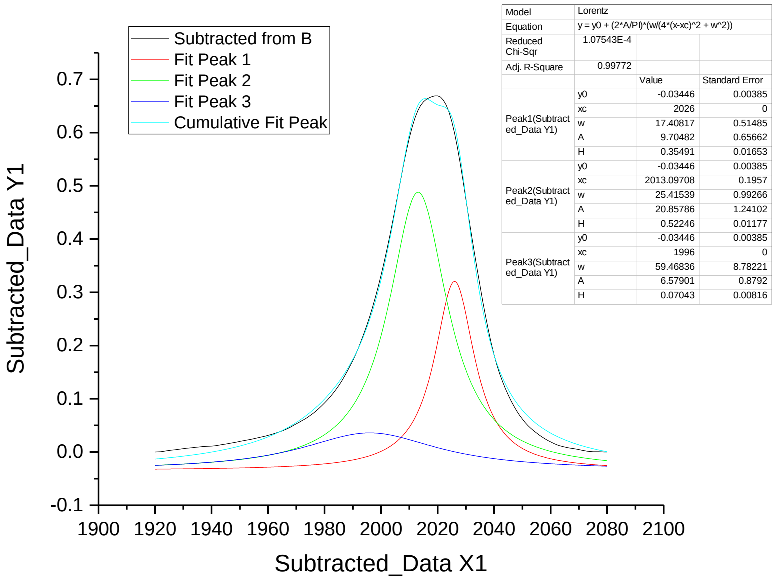

Figure S11. Peak fitting analysis of three consecutive IR spectra acquired during the oxidation of $[Rh_{21}Sb_2(CO)_{38}]^{5-}$.

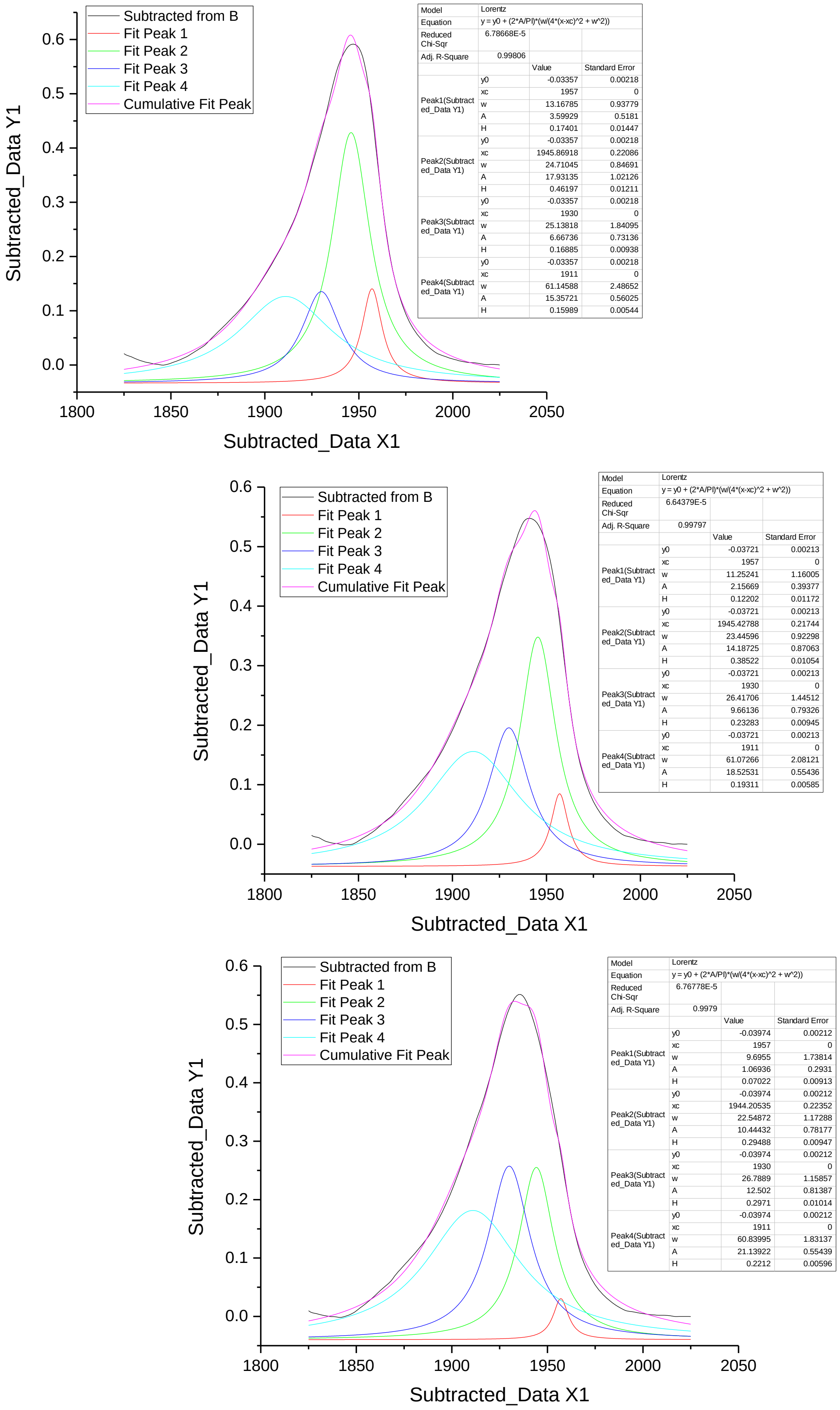

Figure S12. Peak fitting analysis of three consecutive IR spectra acquired during the reduction of $[Rh_{21}Sb_2(CO)_{38}]^{5-}$ from –1.90 to –2.1 V *vs.* Ag pseudoreference electrode.

IR and ^{31}P NMR spectra for $[Rh_{10}Sb(CO)_{21}PPh_3]^{3-}$ (4).

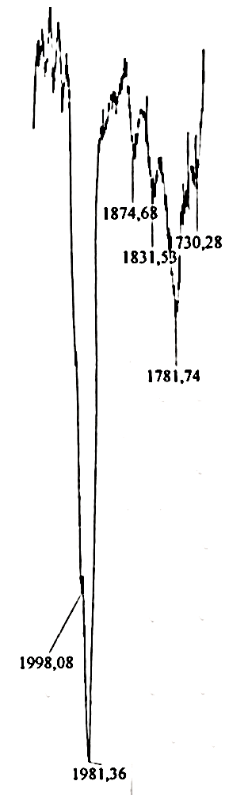

Figure S13. IR spectrum of $[Rh_{10}Sb(CO)_{21}PPh_3][NEt_4]_3 \cdot CH_3CN$ registered in CH_3CN solution.

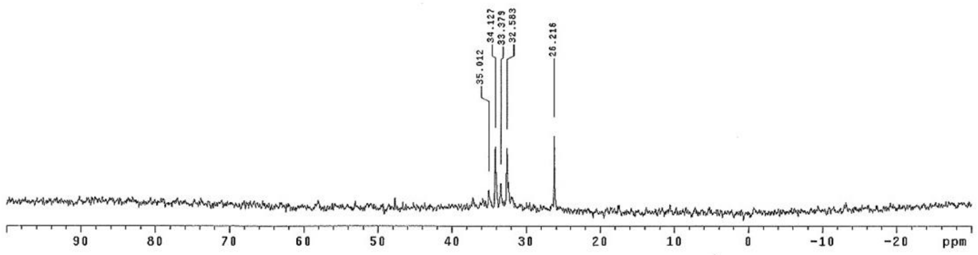

Figure S14. ^{31}P NMR spectrum of $[Rh_{10}Sb(CO)_{21}PPh_3]^{3-}$ in CD_3CN at 298 K.

Most relevant bond distances for $[Rh_{20}Sb_3(CO)_{36}]^{3-}$ (1).

Sb(1)-Rh(6)	2.532(3)
Sb(1)-Rh(16)	2.615(3)
Sb(1)-Rh(20)	2.616(3)
Sb(1)-Rh(18)	2.624(3)
Sb(1)-Rh(8)	2.696(3)
Sb(1)-Rh(11)	2.698(3)
Sb(1)-Rh(9)	2.920(3)
Sb(1)-Rh(14)	2.925(3)
Sb(1)-Sb(2)	3.014(3)
Sb(1)-Rh(4)	3.052(3)
Sb(1)-Rh(13)	3.064(3)
Sb(2)-Rh(10)	2.656(3)
Sb(2)-Rh(7)	2.663(3)
Sb(2)-Rh(6)	2.716(3)
Sb(2)-Rh(8)	2.834(3)
Sb(2)-Rh(11)	2.845(3)
Sb(2)-Rh(4)	3.071(3)
Sb(2)-Rh(13)	3.076(3)
Sb(3)-Rh(6)	2.573(3)
Sb(3)-Rh(17)	2.653(3)
Sb(3)-Rh(22)	2.669(3)
Sb(3)-Rh(23)	2.682(3)
Sb(3)-Rh(21)	2.683(3)
Sb(3)-Rh(19)	2.694(3)
Sb(3)-Rh(7)	2.886(3)
Sb(3)-Rh(10)	2.887(3)
Sb(3)-Rh(15)	2.925(3)
Sb(3)-Rh(12)	2.951(3)
Sb(3)-Rh(5)	2.964(3)
Rh(4)-Rh(5)	2.744(3)
Rh(4)-Rh(10)	2.775(3)
Rh(4)-Rh(6)	2.817(3)
Rh(4)-Rh(18)	2.895(3)
Rh(4)-Rh(8)	2.910(4)
Rh(4)-Rh(14)	3.104(3)
Rh(5)-Rh(14)	2.793(3)
Rh(5)-Rh(6)	2.832(4)
Rh(5)-Rh(19)	2.871(3)
Rh(5)-Rh(17)	2.940(3)
Rh(5)-Rh(12)	2.954(3)
Rh(5)-Rh(10)	3.150(3)

Rh(6)-Rh(9)	2.801(3)
Rh(6)-Rh(14)	2.816(3)
Rh(6)-Rh(13)	2.821(4)
Rh(6)-Rh(15)	2.833(3)
Rh(6)-Rh(12)	2.842(3)
Rh(6)-Rh(10)	2.871(3)
Rh(6)-Rh(7)	2.882(3)
Rh(7)-Rh(13)	2.769(3)
Rh(7)-Rh(22)	2.869(3)
Rh(7)-Rh(21)	2.881(3)
Rh(7)-Rh(10)	3.012(3)
Rh(7)-Rh(15)	3.125(3)
Rh(8)-Rh(11)	2.888(3)
Rh(8)-Rh(18)	2.914(3)
Rh(9)-Rh(12)	2.806(3)
Rh(9)-Rh(15)	2.807(3)
Rh(9)-Rh(14)	2.918(3)
Rh(9)-Rh(16)	2.921(4)
Rh(9)-Rh(20)	2.951(3)
Rh(9)-Rh(13)	3.090(3)
Rh(10)-Rh(17)	2.854(4)
Rh(10)-Rh(22)	2.883(3)
Rh(11)-Rh(13)	2.907(3)
Rh(11)-Rh(16)	2.914(3)
Rh(12)-Rh(14)	2.826(3)
Rh(12)-Rh(19)	2.892(3)
Rh(12)-Rh(23)	2.901(3)
Rh(12)-Rh(15)	2.945(4)
Rh(13)-Rh(15)	2.738(3)
Rh(13)-Rh(16)	2.908(3)
Rh(14)-Rh(20)	2.928(3)
Rh(14)-Rh(18)	2.945(3)
Rh(15)-Rh(23)	2.876(4)
Rh(15)-Rh(21)	2.920(3)
Rh(16)-Rh(20)	2.909(4)
Rh(17)-Rh(19)	2.856(3)
Rh(17)-Rh(22)	2.923(4)
Rh(18)-Rh(20)	2.915(3)
Rh(19)-Rh(23)	2.860(3)
Rh(21)-Rh(23)	2.861(3)
Rh(21)-Rh(22)	2.932(3)

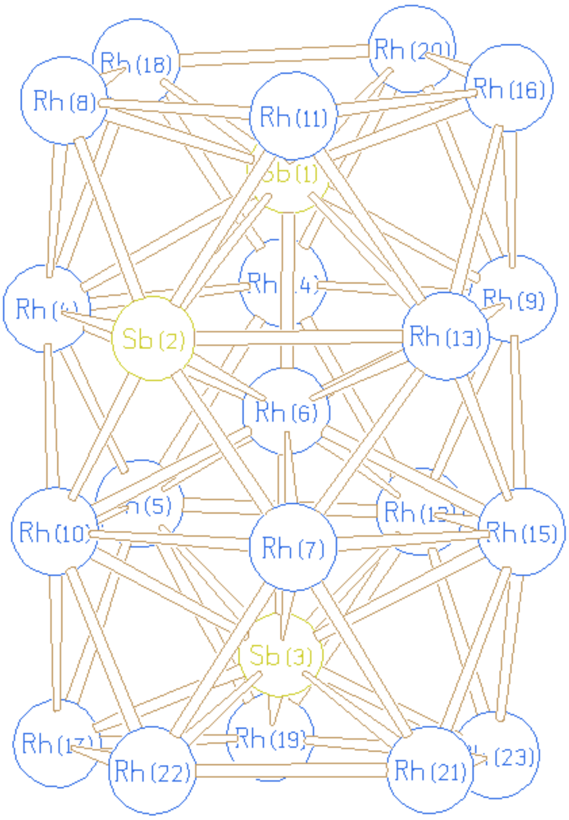

Rh(18)
Rh(20)
Rh(8)
Rh(11)
Rh(16)
Rh(9)
Sb(2)
Rh(13)
Rh(6)
Rh(10)
Rh(7)
Rh(15)
Sb(3)
Rh(19)
Rh(22)
Rh(21)

Most relevant bond distances for $[Rh_{21}Sb_2(CO)_{38}]^{5-}$ (3), first isomer.

Sb(1)-Rh(2)	2.5128(9)
Sb(1)-Rh(9)	2.6471(11)
Sb(1)-Rh(10)	2.6529(13)
Sb(1)-Rh(12)	2.6583(10)
Sb(1)-Rh(11)	2.6731(10)
Sb(1)-Rh(8)	2.6972(10)
Sb(1)-Rh(4)#1	2.9005(13)
Sb(1)-Rh(7)#1	2.9010(14)
Sb(1)-Rh(6)#1	2.9097(11)
Sb(1)-Rh(3)#1	2.9242(11)
Sb(1)-Rh(5)	2.9389(10)
Rh(2)-Sb(1)#1	2.5128(9)
Rh(4)-Sb(1)#1	2.9004(13)
Rh(3)-Sb(1)#1	2.9242(11)
Rh(6)-Sb(1)#1	2.9096(11)
Rh(7)-Sb(1)#1	2.9010(14)
Rh(2)-Rh(7)	2.7866(9)
Rh(2)-Rh(7)#1	2.7866(9)
Rh(2)-Rh(3)#1	2.8024(11)
Rh(2)-Rh(3)	2.8024(11)
Rh(2)-Rh(6)#1	2.8114(10)
Rh(2)-Rh(4)#1	2.8116(11)
Rh(2)-Rh(6)	2.8113(10)
Rh(2)-Rh(4)	2.8115(11)
Rh(2)-Rh(5)#1	2.8307(12)
Rh(2)-Rh(5)	2.8307(12)
Rh(3)-Rh(5)	2.7733(12)
Rh(3)-Rh(4)#1	2.7970(11)
Rh(3)-Rh(9)#1	2.8898(11)
Rh(3)-Rh(8)#1	2.9298(13)
Rh(3)-Rh(7)	2.9393(12)
Rh(3)-Rh(6)	3.0535(12)

Rh(4)-Rh(7)#1	2.7063(10)
Rh(4)-Rh(3)#1	2.7971(11)
Rh(4)-Rh(10)#1	2.8696(12)
Rh(4)-Rh(12)#1	2.9015(11)
Rh(4)-Rh(6)	2.9445(12)
Rh(4)-Rh(5)#1	3.0910(11)
Rh(5)-Rh(6)	2.7880(11)
Rh(5)-Rh(11)	2.8712(15)
Rh(5)-Rh(12)	2.8830(13)
Rh(5)-Rh(7)#1	3.0705(10)
Rh(5)-Rh(4)#1	3.0910(12)
Rh(6)-Rh(7)#1	2.7917(14)
Rh(6)-Rh(10)#1	2.8977(10)
Rh(6)-Rh(8)#1	2.9172(15)
Rh(7)-Rh(4)#1	2.7063(10)
Rh(7)-Rh(6)#1	2.7916(14)
Rh(7)-Rh(9)#1	2.8552(12)
Rh(7)-Rh(11)#1	2.9047(10)
Rh(7)-Rh(5)#1	3.0705(10)
Rh(8)-Rh(9)	2.8734(11)
Rh(8)-Rh(10)	2.8781(10)
Rh(8)-Rh(6)#1	2.9172(15)
Rh(8)-Rh(3)#1	2.9298(13)
Rh(9)-Rh(7)#1	2.8552(12)
Rh(9)-Rh(11)	2.8783(12)
Rh(9)-Rh(3)#1	2.8898(11)
Rh(10)-Rh(4)#1	2.8696(12)
Rh(10)-Rh(12)	2.8706(12)
Rh(10)-Rh(6)#1	2.8977(10)
Rh(11)-Rh(12)	2.8745(12)
Rh(11)-Rh(7)#1	2.9047(11)
Rh(12)-Rh(4)#1	2.9015(11)

Most relevant bond distances for $[Rh_{21}Sb_2(CO)_{38}]^{5-}$ (3), second isomer.

Sb(21)-Rh(22)	2.5136(9)
Sb(21)-Rh(29)	2.6487(9)
Sb(21)-Rh(30)	2.6529(13)
Sb(21)-Rh(27)	2.6570(11)
Sb(21)-Rh(31)	2.6588(9)
Sb(21)-Rh(32)	2.6844(11)
Sb(21)-Rh(23)#2	2.8919(10)
Sb(21)-Rh(28)#2	2.9001(10)
Sb(21)-Rh(26)#2	2.9066(14)
Sb(21)-Rh(24)#2	2.9206(12)
Sb(21)-Rh(25)	2.9570(13)
Rh(22)-Sb(21)#2	2.5136(9)
Rh(23)-Sb(21)#2	2.8918(10)
Rh(24)-Sb(21)#2	2.9206(12)
Rh(26)-Sb(21)#2	2.9066(14)
Rh(28)-Sb(21)#2	2.9000(10)
Rh(22)-Rh(28)	2.7881(12)
Rh(22)-Rh(28)#2	2.7882(11)
Rh(22)-Rh(23)	2.7923(11)
Rh(22)-Rh(23)#2	2.7924(11)
Rh(22)-Rh(24)#2	2.8182(10)
Rh(22)-Rh(24)	2.8183(10)
Rh(22)-Rh(26)#2	2.8193(11)
Rh(22)-Rh(26)	2.8193(11)
Rh(22)-Rh(25)	2.8263(10)
Rh(22)-Rh(25)#2	2.8263(10)
Rh(23)-Rh(28)#2	2.7200(10)
Rh(23)-Rh(24)#2	2.7902(14)
Rh(23)-Rh(27)#2	2.8775(13)
Rh(23)-Rh(31)#2	2.8902(15)
Rh(23)-Rh(26)	2.9437(11)
Rh(23)-Rh(25)#2	3.0615(12)

Rh(24)-Rh(25)	2.7715(12)
Rh(24)-Rh(23)#2	2.7903(14)
Rh(24)-Rh(29)#2	2.8912(12)
Rh(24)-Rh(32)#2	2.9139(12)
Rh(24)-Rh(28)	2.9580(12)
Rh(24)-Rh(26)	3.0747(11)
Rh(25)-Rh(26)	2.7914(11)
Rh(25)-Rh(30)	2.8815(12)
Rh(25)-Rh(27)	2.8854(13)
Rh(25)-Rh(23)#2	3.0616(13)
Rh(25)-Rh(28)#2	3.0630(13)
Rh(26)-Rh(28)#2	2.7857(13)
Rh(26)-Rh(31)#2	2.8956(10)
Rh(26)-Rh(32)#2	2.9084(11)
Rh(27)-Rh(30)	2.8476(11)
Rh(27)-Rh(31)	2.8669(11)
Rh(27)-Rh(23)#2	2.8775(13)
Rh(28)-Rh(23)#2	2.7201(10)
Rh(28)-Rh(26)#2	2.7857(13)
Rh(28)-Rh(29)#2	2.8696(15)
Rh(28)-Rh(30)#2	2.9008(11)
Rh(28)-Rh(25)#2	3.0629(13)
Rh(29)-Rh(28)#2	2.8696(15)
Rh(29)-Rh(30)	2.8717(11)
Rh(29)-Rh(32)	2.8779(12)
Rh(29)-Rh(24)#2	2.8912(12)
Rh(30)-Rh(28)#2	2.9010(11)
Rh(31)-Rh(32)	2.8769(13)
Rh(31)-Rh(23)#2	2.8902(15)
Rh(31)-Rh(26)#2	2.8955(10)
Rh(32)-Rh(26)#2	2.9085(12)
Rh(32)-Rh(24)#2	2.9139(12)

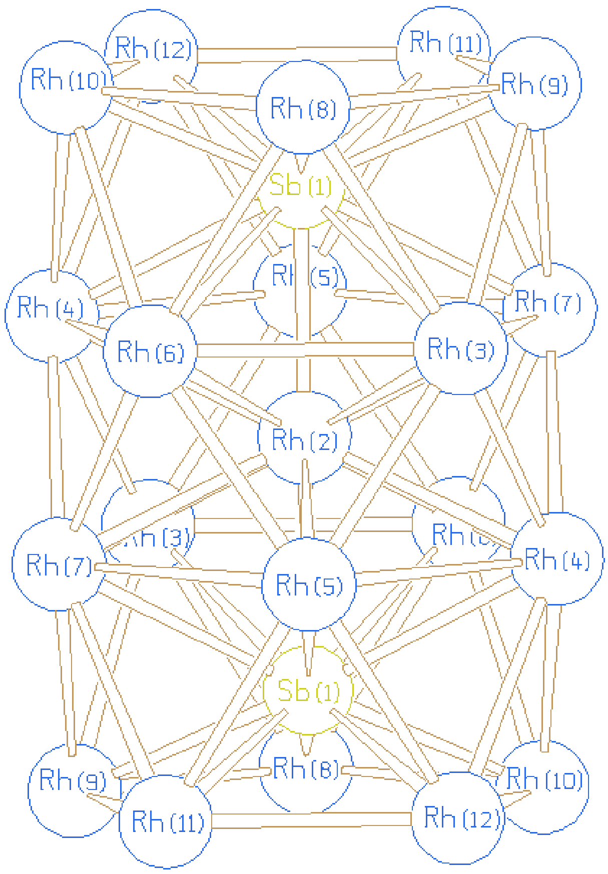
Rh(12)
Rh(11)
Rh(10)
Rh(8)
Rh(9)
Sb(1)
Rh(4)
Rh(7)
Rh(6)
Rh(3)
Rh(2)
Rh(7)
Rh(5)
Rh(4)
Sb(1)
Rh(9)
Rh(8)
Rh(10)
Rh(11)
Rh(12)

Most relevant bond distances for $[Rh_{10}Sb(CO)_{21}PPh_3]^{3-}$ (4).

Sb(1)-Rh(2)	2.6189(3)
Sb(1)-Rh(6)	2.6862(3)
Sb(1)-Rh(3)	2.6878(3)
Sb(1)-Rh(7)	2.6967(3)
Sb(1)-Rh(11)	2.7370(3)
Sb(1)-Rh(4)	2.7503(3)
Sb(1)-Rh(9)	2.7653(3)
Sb(1)-Rh(8)	2.7672(3)
Sb(1)-Rh(5)	2.7683(3)
Sb(1)-Rh(10)	2.7870(3)
Rh(2)-Rh(7)	2.8345(3)
Rh(2)-Rh(5)	2.8714(4)
Rh(2)-Rh(8)	3.0568(4)
Rh(3)-Rh(6)	2.8246(4)
Rh(3)-Rh(4)	2.8715(4)
Rh(3)-Rh(5)	2.8805(3)

Rh(3)-Rh(9)	3.0286(4)
Rh(4)-Rh(9)	2.9051(4)
Rh(4)-Rh(11)	2.9269(4)
Rh(4)-Rh(6)	2.9983(4)
Rh(4)-Rh(10)	3.0526(4)
Rh(5)-Rh(8)	2.9407(4)
Rh(5)-Rh(9)	2.9999(4)
Rh(6)-Rh(11)	2.8337(4)
Rh(7)-Rh(8)	2.8697(4)
Rh(7)-Rh(11)	2.8935(4)
Rh(7)-Rh(10)	2.9842(4)
Rh(8)-Rh(10)	2.8999(4)
Rh(8)-Rh(9)	3.0011(4)
Rh(9)-Rh(10)	2.8579(4)
Rh(10)-Rh(11)	3.0291(4)

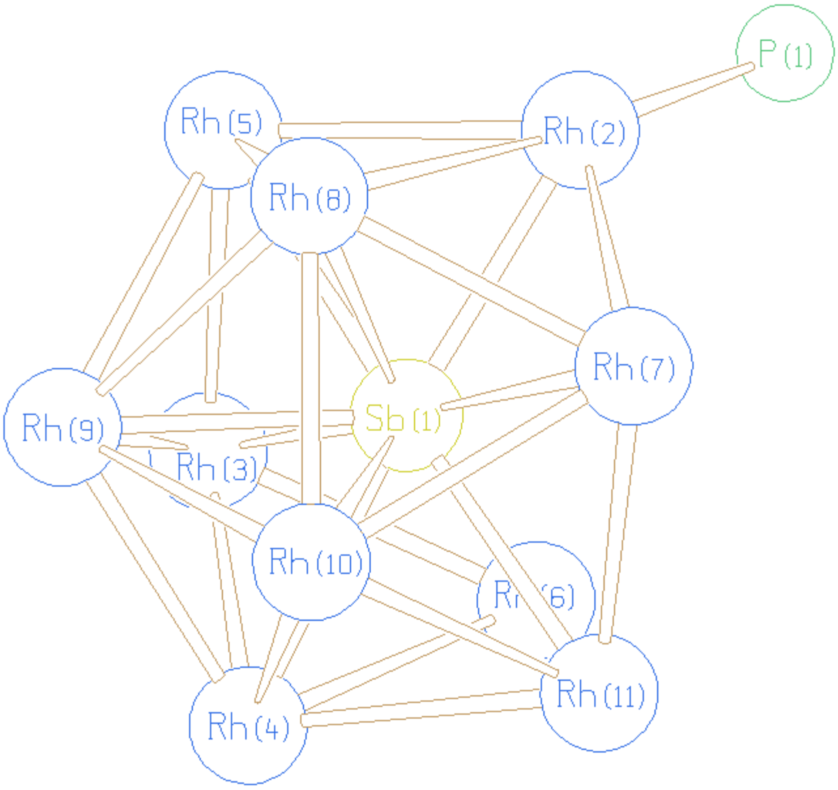

Supplement: Supplementary file 1 — ic9b03135_si_001.pdf [file ic9b03135_si_001.pdf]
